# The macrophage microtubule network acts as a key cellular controller of the intracellular fate of *Leishmania infantum*

**DOI:** 10.1371/journal.pntd.0008396

**Published:** 2020-07-28

**Authors:** Sandrine Cojean, Valérie Nicolas, Vanessa Lievin-Le Moal

**Affiliations:** 1 Université Paris-Saclay, CNRS, UMR 8076 BioCis, Châtenay-Malabry, France; 2 Université Paris-Saclay, Institut Paris-Saclay d’Innovation Thérapeutique (UMS-IPSIT), Unité Mixte de Services, Microscopy Facility, Châtenay-Malabry, France; 3 Université Paris-Saclay, Inserm, UMR-S 996 Inflammation, Microbiome and Immunosurveillance, Clamart, France; University of São Paulo FMRP/USP, BRAZIL

## Abstract

The parasitophorous vacuoles (PVs) that insulate *Leishmania spp*. in host macrophages are vacuolar compartments wherein promastigote forms differentiate into amastigote that are the replicative form of the parasite and are also more resistant to host responses. We revisited the biogenesis of tight-fitting PVs that insulate *L*. *infantum* in promastigote-infected macrophage-like RAW 264.7 cells by time-dependent confocal laser multidimensional imaging analysis. Pharmacological disassembly of the cellular microtubule network and silencing of the dynein gene led to an impaired interaction of *L*. *infantum*-containing phagosomes with late endosomes and lysosomes, resulting in the tight-fitting parasite-containing phagosomes never transforming into mature PVs. Analysis of the shape of the *L*. *infantum* parasite within PVs, showed that factors that impair promastigote-amastigote differentiation can also result in PVs whose maturation is arrested. These findings highlight the importance of the MT-dependent interaction of *L*. *infantum*-containing phagosomes with the host macrophage endolysosomal pathway to secure the intracellular fate of the parasite.

## Author summary

Kinetoplastid parasites of the genus *Leishmania* are responsible for a diverse spectrum of mammalian infectious diseases, the leishmaniases, including cutaneous, mucocutaneous, and mucosal pathologies. Infectious metacyclic promastigotes of infected female Phlebotomus sandflies are injected into the host at the site of the bite during the sandfly blood meal, after which they are internalized by host professional phagocytic neutrophils and macrophages. *Leishmania infantum* is an etiological agent of potentially fatal visceral pathology. This study molecularly dissects the maturation of *L*. *infantum*-containing phagosomes/parasitophorous vacuoles (PVs) in host macrophages. We reveal the requirement of vacuolar movement along macrophage microtubule tracks for the phagosome trafficking toward the endolysosomal pathway necessary for the development of the mature tight-fitting PV crucial for *L*. *infantum* survival and proliferation.

## Introduction

Leishmaniasis, the second leading cause of death due to parasitic diseases in the world after malaria, is the most significant of the neglected tropical diseases, affecting approximately 12 million people, with 350 million at risk in 98 countries worldwide [1,2]. *Leishmania* is amongst the protozoan parasites that exhibit an obligate intracellular lifestyle [3,4]. Infecting promastigotes are internalized by target host neutrophils, dendritic cells, and macrophages recruited to the site of the sand fly bite [5]. In macrophages, after closure of the phagocytic cup to form a phagosome that insulates one promastigote, the parasites construct two forms of replication-competent vacuolar niches, depending on the *Leishmania* species [3,4]. The tight-fitting parasitophorous vacuole (PV) insulates one parasite for species of the *L*. *donovani* complex and *L*. *major* and a very large communal PV insulates numerous parasites for species of the *L*. *mexicana* complex [3,4]. Parasite-containing PVs exhibit a hybrid nature, including several functional characteristics of phagolysosomes [6], as the result of identical successive heterotypic fusions with host-cell late endosomes (LEs) and lysosomes of the endolysosomal pathway and endoplasmic reticulum (ER)-derived vacuoles [3,4]. Remarkably, the insulated, infective *Leishmania* promastigotes trigger sophisticated virulence factor-dependent mechanisms that principally retard the maturation of early phagosomes to PVs by delaying their fusion with LEs and lysosomes [7,8]. Moreover, *Leishmania* have developed the capacity to remodel the PV membrane [9–12]. In addition, insulated promastigotes engage in morphological and functional differentiation into amastigotes, which are particularly well adapted to live in the degradative luminal environment of the mature PVs.

Development of the physical and dynamic association of *Leishmania spp*.-containing PVs with vacuolar compartments of the host cell endocytic pathway [3,4] has not been completely elucidated. Similarities are with the phagolysosome biogenesis in which early phagosomes are converted into late phagosomes through their fusion with LEs and then late phagosomes become phagolysosomes through their fusion with lysosomes [6]. These vacuolar fusion events follow physical vacuolar interactions controlled by dynein-dependent vacuolar movements along microtubule (MT) tracks [6]. We thus studied the role of MT tracks in the intramacrophage lifestyle of *Leishmania spp*. As the infecting species, we used *L*. *infantum* (formerly named *L*. *chagasi* or *L*. *infantum chagasi*), which is the main causative agent of zoonotic visceral leishmaniasis and also responsible for cases of cutaneous leishmaniasis in the Mediterranean Basin, including southern Europe, northern Africa, and parts of Asia [13]. By affecting internal organs, such as the liver, spleen, and bone marrow, *L*. *infantum* is the most severe form of *Leishmania*-triggered diseases, causing death if left untreated [14]. We show that microtubule (MT)-dependent routing of the *L*. *infantum*-containing phagosomes towards LEs and lysosomes is pivotal for maturation of the insulating phagosomes into tight-fitting PVs. In addition, we provide evidence that PV maturation is necessary for the insulated parasites to completely differentiate into the resistant amastigote form that is able to survive and replicate.

## Results

### Differentiation of the body shape of *L*. *infantum* LEM 5700 hosted in macrophage-like RAW 264.7 cells

**Fig 1:**
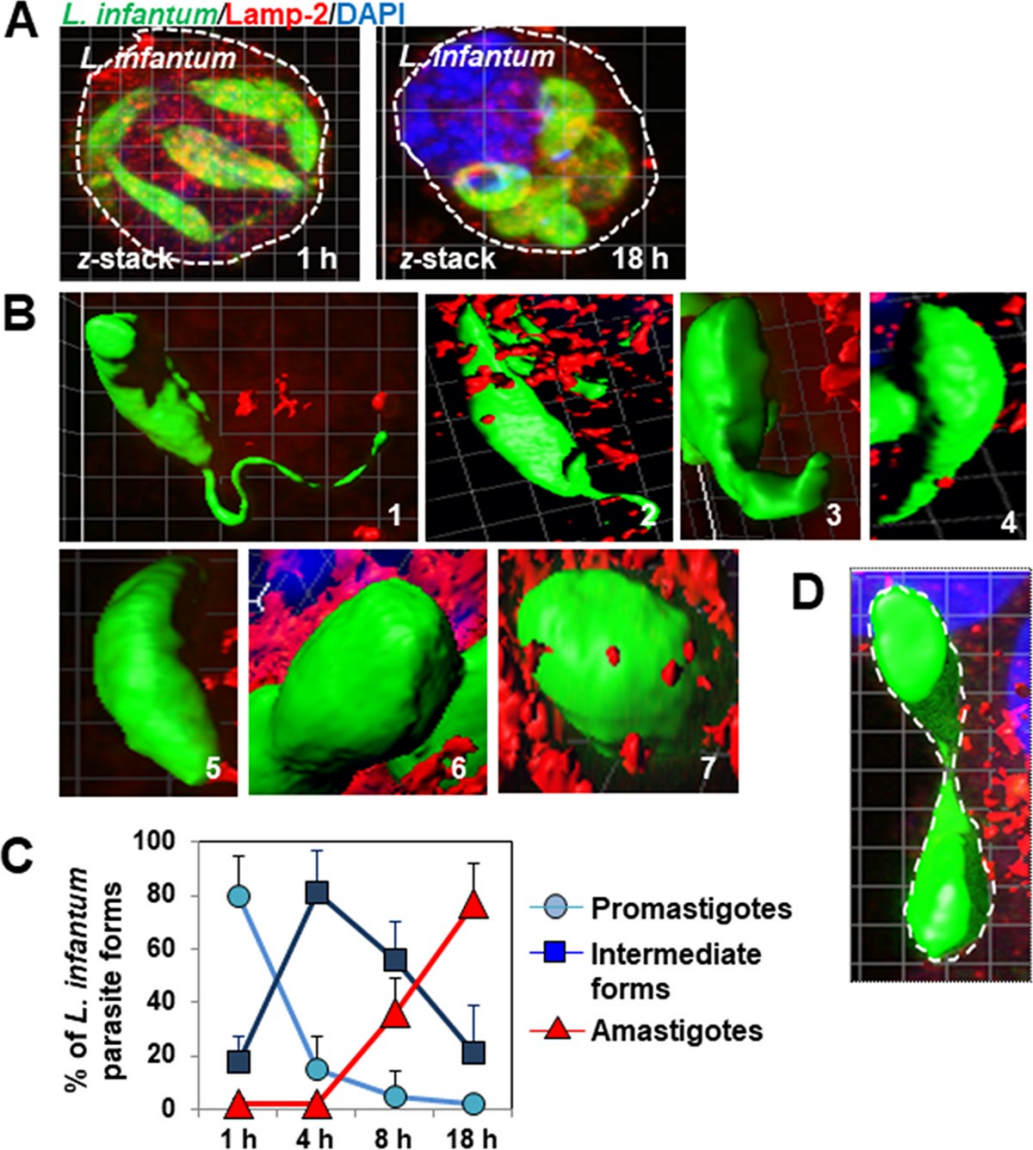
Time-course of promastigote-to-amastigote differentiation of *L*. *infantum* LEM 5700 parasites hosted in macrophage-like RAW 264.7 cells. **(A)** Confocal micrographs showing intracellular promastigotes (left image) and amastigotes (right image) in macrophage-like cells. **(B)** 3D-reconstruction surface rendering confocal micrographs of the parasite body shape transformation during an infection time-course. 1—A promastigote form expressing one long flagellum positioned at the anterior pole of the spindle-shaped body. 2—Fusiform-like form with a shortened flagellum. 3—Condensed fusiform-like form with an enlarged anterior appendage. 4—Condensed fusiform-like form engulfing the enlarged appendage. 5—Non-flagellated condensed ellipsoidal form. 6—Non-flagellated highly condensed ovoid form. 7—Non-flagellated round amastigote form. **(C)** Quantification of the number of promastigotes, intermediate differentiated forms (summation of parasitic forms viewed in Fig 2 to 6), and amastigotes during an infection time-course. **(D)** 3D-reconstruction surface rendering confocal micrograph showing elongation of an *L*. *infantum*-containing vacuole preceding fission into two new parasite-containing vacuoles. The white dashed box shows the elongated *L*. *infantum*-containing vacuole viewed at high magnification in the adjacent image (Right panel). The white dashed line delineates the area of the elongated vacuole. Confocal micrographs are representative of two independent experiments in duplicate. The percentage of parasite forms was determined by examining at least 30 cells at each time-point for each condition. Data are presented as averages ± SEM. Data are from two or three independent experiments in duplicate.

*L*. *infantum* LEM 5700 promastigotes infected macrophage-like RAW 264.7 cells in which they differentiated into amastigotes (Fig 1A). 3D-reconstruction surface rendering confocal micrographs made it possible, for the first time, to follow the successive transformation of body shape of *L*. *infantum* hosted in macrophage-like RAW 264.7 cells during a time-course of infection. At early time-points PI, promastigotes had a typical spindle-shaped body, with one flagellum positioned at their anterior pole (Fig 1B, micrograph 1). The first observable morphological change was shortening of the flagellum, without modification of the spindle-shaped body (Fig 1B, micrograph 2). Next, the shortened flagellum transformed into an enlarged appendage (Fig 1B, micrograph 3) that finally disappeared into the parasite body, along with condensation of the spindle-shaped body itself (Fig 1B, micrographs 4 and 5). The condensed ellipsoidal form then transformed into a highly-condensed ovoid form (Fig 1B, micrograph 6), which ultimately developed into the spherical amastigote form (Fig 1B, micrograph 7). We then quantified the time-dependent evolution of the *L*. *infantum* forms arbitrarily grouped into three types: undifferentiated promastigotes, intermediate forms engaged in differentiation, and highly differentiated amastigotes (Fig 1C). There was rapid disappearance of the promastigotes from 1 h to 4 h PI, accompanied by the rapid appearance of the intermediate parasite forms. Subsequently, the number of intermediate forms steadily decreased from 4 to 18 h PI, at the same time as the number of amastigotes increased. In addition, CLSM showed the replication of *L*. *infantum* amastigotes, in which the PV membrane elongated preceding its fission, generating two new tight-fitting PVs, each containing one progeny amastigote (Fig 1D).

### Biogenesis of tight-fitting *L*. *infantum* LEM 5700-containing PVs hosted in macrophage-like RAW 264.7 cells

Wilson and co-workers [15–18] showed the decoration of *L*. *infantum chagasi*-containing vacuoles by several bona fide markers of sub-compartments of the host-cell endolysosomal pathway at various times post-infection (PI) in promastigote-infected human monocyte-derived macrophages, human U937 monocytic cells, and mouse bone-marrow-derived macrophages. This included transient decoration by early endosome antigen-1 and Rab5 GTPase and stable decoration by membrane-associated protein-1, belonging to the Lamp1/2 family of proteins distributed among LEs and lysosomes [19]. We revisited and extended these observations by conducting a time-course experiment to assess and quantify the association of a large set of endolysosomal markers with the parasites insulated in mouse macrophage-like RAW 264.7 cells infected with *L*. *infantum* promastigotes. We examined the classical markers, membrane-associated Rab7 GTPase, a marker of the transition between early endosomes (EEs) and LEs [20], membrane-associated protein-2 (Lamp-2) [19], and cathepsin D (CatD), an aspartyl protease that represents a major category of luminal lysosomal hydrolases [21]. In addition, we followed the Qb-SNAREs (soluble N‐ethylmaleimide‐sensitive factor attachment protein receptors) Vti1a and Vti1b (vesicle transport through t-SNARE Interaction) [22]. Knowledge of the membrane composition of tight-fitting PVs that insulate species of the *L*. *donovani* complex and *L*. *major* in terms of functional proteins is limited [9–12]. We also examined, for the first time, the lysosomal membrane-associated class III glucose/fructose transporter, GLUT8 (*Slc2A8*) [23,24] and polypeptide transporter associated with antigen processing-like (TAPL; *ABCB9*) [24,25].

**Fig 2:**
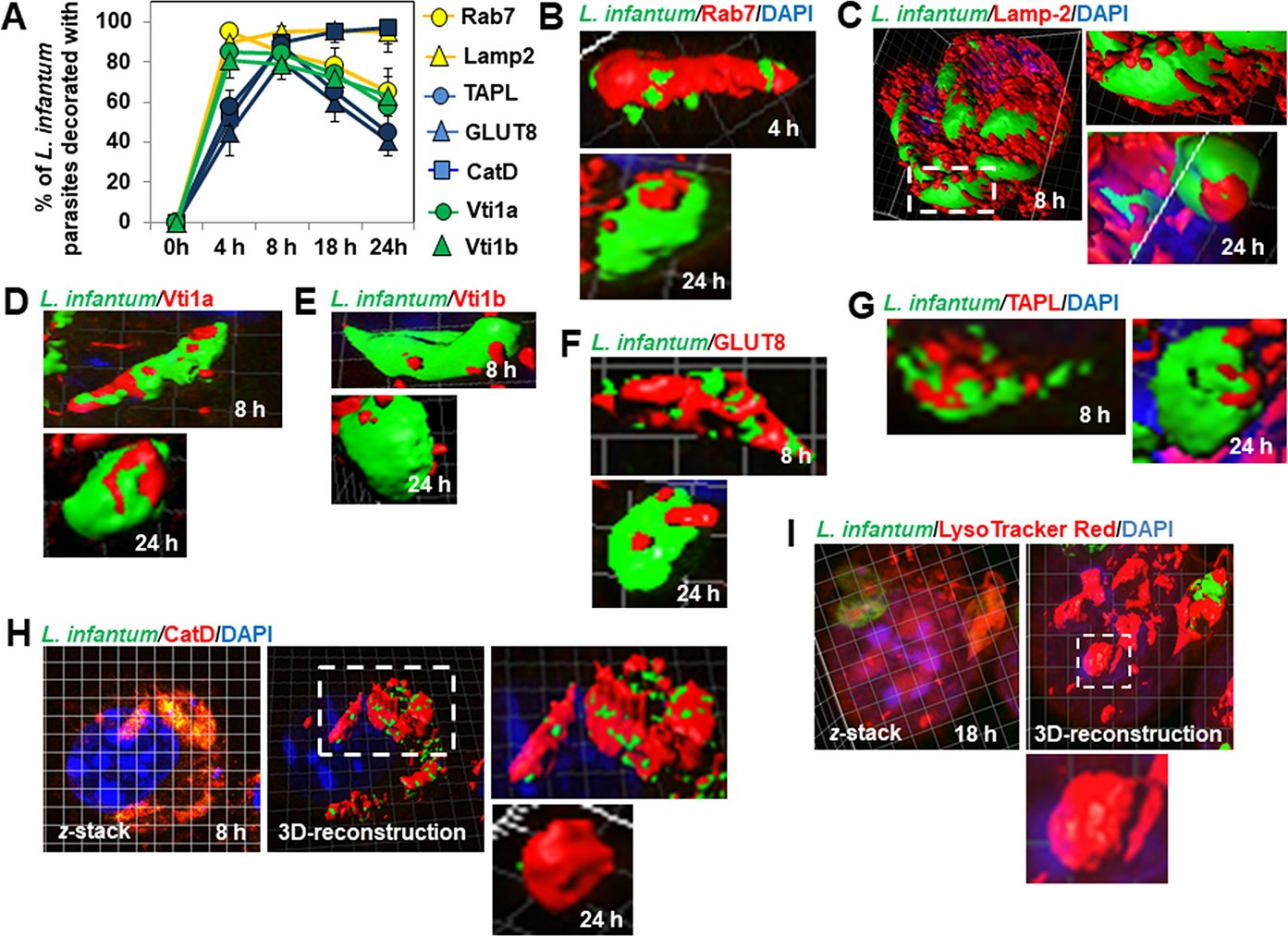
Decoration of hosted *L*. *infantum* LEM 5700 parasites by the GTPase Rab7, Qb-SNAREs Vti1a and Vti1b, Lamp-2, hydrolase cathepsin D, hexose transporter GLUT8, and polypeptide transporter TAPL during an infection time-course in macrophage-like RAW 264.7 cells. (**A**) Graph showing the kinetics of decoration of parasites by Rab7, Lamp-2, cadherin D (CatD), GLUT8, TAPL, Vti1a, and Vti1b during an infection time-course. **(B)** 3D-reconstruction surface rendering confocal micrographs of a fusiform-like parasite and an amastigote decorated with Rab7-positive patches. **(C)** 3D-reconstruction surface rendering confocal micrographs of fusiform-like and ellipsoidal parasite forms and amastigotes densely decorated with elongated Lamp-2-positive lamellae and Lamp-2-positive patches. See also S1 Video. **(D)** 3D-reconstruction surface rendering confocal micrographs of a fusiform-like parasite decorated by large Vti1a-positive patches and an amastigote sparsely decorated with large Vti1a-positive patches. **(E)** 3D-reconstruction surface rendering confocal micrographs of a fusiform-like parasite decorated by small Vti1b-positive patches and an amastigote sparsely decorated with small Vti1b-positive patches. **(F)** 3D-reconstruction surface rendering confocal micrographs of a fusiform-like parasite highly decorated with GLUT8-positive large patches and an amastigote sparsely decorated with small GLUT8-positive patches. **(G)** 3D-reconstruction surface rendering confocal micrographs of a fusiform-like parasite decorated with TAPL-positive patches and an amastigote sparsely decorated with small TAPL-positive patches. **(H)** *z*-stack and 3D-reconstruction surface rendering confocal micrographs of fusiform-like and ellipsoidal parasite forms, and amastigotes almost completely enwrapped by large and continuous CatD-positive patches. See also S2 Video. (I) *z*-stack and 3D-reconstruction surface rendering confocal micrographs showing strong Lysotracker Red positivity for ellipsoidal, ovoid, and round parasite forms. Confocal micrographs are representative of three independent experiments. The percentage of positive parasites for each endolysosomal marker was determined by counting parasites hosted in at least 30 cells for each time-point. Data are represented as the average ± SEM.

Multi-channel confocal laser scanning microscopy (CLSM) acquisition and 3D-reconstruction surface rendering allowed the simultaneous observation of immunolabeled endolysosomal markers and parasites in infected cells. Quantification of decorated intramacrophage parasites during a time-course PI (Fig 2A) showed a rapid increase in the percentage of Rab7-positive parasites, reaching a maximum at 4 h PI, which than tended to decrease. The percentage of parasites decorated with Lamp-2 increased rapidly, reaching a maximum at 4 h PI, and remained stable up to 24 h. The percentage of parasites positive for Vti1a and Vti1b increased rapidly, reaching a maximum at 4 h PI, and then declined significantly. The percentage of CatD-positive parasites increased more slowly, reaching a maximum at 8 h PI, and remained stable at the subsequent time-points. We observed that the percentage of GLUT8- and TAPL-positive *L*. *infantum* parasites increased similarly, reaching a maximum at 8 h PI, and then decreased significantly.

3D-reconstruction surface rendering confocal micrographs and videos showed various patterns of decoration of insulated *L*. *infantum* parasites by endolysosomal markers, including randomly distributed small patches of various sizes and large patches wrapping densely or almost entirely around the parasite body. Fusiform-like parasites were decorated by large Rab7-positive patches, whereas amastigotes were only decorated by small patches (Fig 2B). Lamp-2-positive tube-like structures and patches densely decorated both fusiform-like parasites and amastigotes (Fig 2C and S1 Video). Large Vti1a-positive patches decorated the fusiform-like parasites and amastigotes (Fig 2D). Both fusiform-like parasites and amastigotes were sparsely decorated by small randomly distributed Vti1b-positive patches (Fig 2E). Large GLUT8- and TAPL-positive patches decorated the fusiform-like parasites, whereas amastigotes were sparsely decorated by small randomly-distributed patches (Fig 2F and 2G, respectively). In contrast, both fusiform-like forms and amastigotes were almost entirely enwrapped by continuous patches positive for the lysosomal marker CatD (Fig 2H and S2 Video). The observed decoration of *L*. *infantum* parasites by Rab7 and Lamp-2 is reminiscent of their previously observed presence in the membrane of tight-fitting *L*. *donovani*-containing PVs [8,26]. The observed decoration of *L*. *infantum* parasites with Rab7, Lamp-2, and CatD is consistent with the previously observed presence of Rab7p, Lamp-1, and cathepsin B in tight-fitting *L*. *major-*containing PVs [27,28]. Loading of infected cells with LysoTracker Red, a small membrane-permeable lysosomotropic molecule that labels acidic vacuolar compartments, showed the intracellular ovoid and round parasite forms of *L*. *infantum* to be strongly enwrapped by LysoTracker Red fluorescence (Fig 2I).

We next measured the size of the luminal space of phagosomes/PVs that insulated the *L*. *infantum* parasites. We took advantage of the CatD immunofluorescence, which almost completely enwrapped the parasites, and which is known to localize to the luminal space of PVs [28]. Measurement of the volume of the parasite body and that of CatD immunolabeling by isosurface rendering analysis showed there to be a small vacuolar space between the parasite body and phagosome/PV membrane (S1 Fig), in accordance with the known tight-fitting nature of the phagosome/PV insulating parasites of the *L*. *donovani* complex and *L*. *major* [3,4].

### Impairment of the maturation of tight-fitting *L*. *infantum* LEM 5700-containing phagosomes/PVs

We developed a pharmacological approach to block the maturation of *L*. *infantum*-containing phagosomes/PVs that occurs through heterotypic fusion with LEs and lysosomes. We used the small trafficking inhibitor Retro-2, known to block ricin and Shiga-toxin trafficking at the EEs-*trans*-Golgi network (TGN) interface [29,30]. For our time-dependent analysis, we took into account the previously [15–18] and above reported rapid insulation of *L*. *infantum* parasites within early tight-fitting phagosomes and subsequent mature tight-fitting PVs, also previously observed with tight-fitting PV-insulated *L*. *major* [27,31] and *L*. *donovani* [7,32]. Moreover, we also accounted for the fact that Retro-2 exerts direct parasiticide activity against axenic *L*. *amazonensis* at concentrations of 10 to 100 μM [33]. We thus infected the cells with *L*. *infantum* LEM 5700 in the continuous presence of the small compound at sub-parasiticide concentrations of 1 to 5 μM. We therefore ensured that both the number of *L*. *infantum* promastigote*-*infected cells and the number of promastigotes internalized within each cell were not modified relative to untreated infected cells (S2 Fig). In addition, we controlled that the continuous presence of Retro-2 at 1 μM promoted the re-localization of the Qa-SNARE syntaxin 5 (Stx5) from its normal perinuclear localization toward the cytoplasm (S3 Fig) previously observed when the small inhibitor blocks the retrograde transport of the toxins [29].

**Fig 3:**
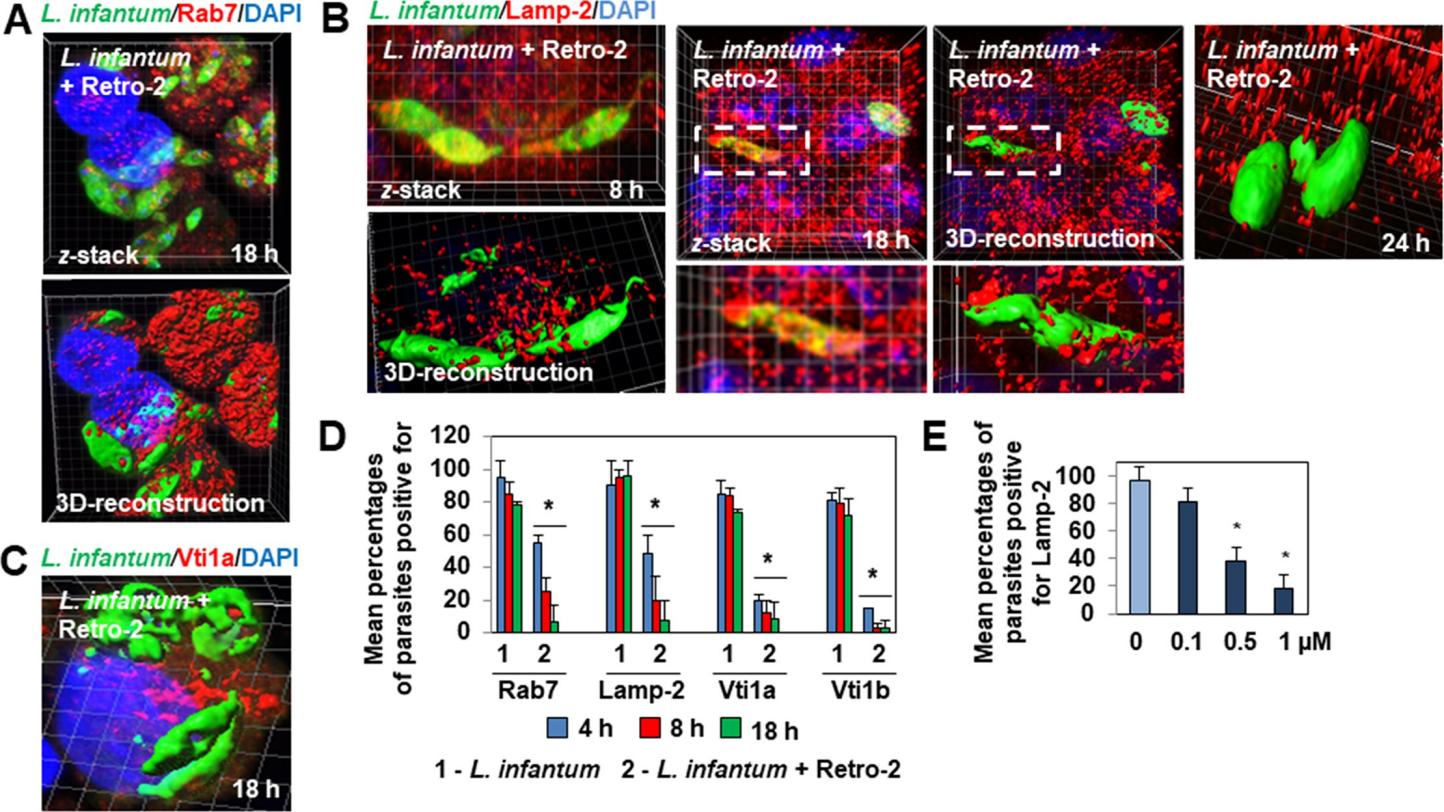
Decrease or lack of decoration of *L*. *infantum* LEM 5700 parasites with GTPase Rab7, Lamp-2, and Qb-SNAREs Vti1a and Vti1b in macrophage-like RAW 264.7 cells infected in the continuous presence of Retro-2. **(A)** *z*-stack and 3D-reconstruction surface rendering confocal micrographs showing decreased decoration of fusiform-like and ellipsoidal parasites hosted in Retro-2 (1 μM)-treated cells with Rab7-positive patches. See Fig 2B for control decoration of parasites in untreated cells. **(B)** *z*-stack and 3D-reconstruction surface rendering confocal micrographs showing decreased decoration of fusiform-like and ellipsoidal parasite forms in Retro-2 (1 μM)-treated infected cells with Lamp-2, characterized by the presence of dispersed Lamp-2-positive small patches. See also S3 Video. See Fig 2C for control decoration of parasites in untreated cells. **(C)** 3D-reconstruction surface rendering confocal micrograph showing the absence of decoration of fusiform-like parasites with Vti1a patches in a Retro-2 (1 μM)-treated infected cell. See Fig 2D for control decoration of parasites in untreated cells. **(D)** Bar graph showing a decrease in the percentage of parasites decorated with Rab7-, Lamp-2-, or Vti1a- or Vti1b-positive patches during an infection time-course in cells infected in the continuous presence of Retro-2 (1 μM). **(E)** Bar graph showing the average percentage of parasites decorated with Lamp-2 in untreated infected cells and cells infected in the continuous presence of increasing concentrations of Retro-2. Confocal micrographs are representative of three independent experiments. High-magnification images correspond to the white dashed boxes. The percentage of parasites decorated with Rab7-, Lamp-2-, Vti1a-, and Vti1b-positive patches was determined by counting parasites hosted in at least 30 cells at each time-point for each condition. Data are represented as the average ± SEM and were analyzed using the unpaired Student t test. *p < 0.01.

3D-reconstruction surface rendering confocal microscopy showed the absence of decoration by the LE-associated Rab7 of the fusiform-like and ellipsoidal forms of the parasites present in 18 h-infected Retro-2-treated cells (Fig 3A and 3D). For Lamp-2, 3D-reconstruction surface rendering confocal microscopy and video showed the absence of decoration of fusiform-like and ellipsoidal parasite forms by Lamp-2-positive tube-like and small patches in 8 h-, 18 h- and 24 h-treated infected cells (Fig 3B and 3D, and S3 Video). Similarly, parasite forms present in 8 h-, 18 h- and 24 h-treated infected cells showed the absence of decoration by Vti1a and Vti1b (Fig 3C and 3D). Quantification of Lamp-2 showed the decreased association of LE-associated markers with the parasites to develop as a function of the concentration of Retro-2 (Fig 3E).

**Fig 4:**
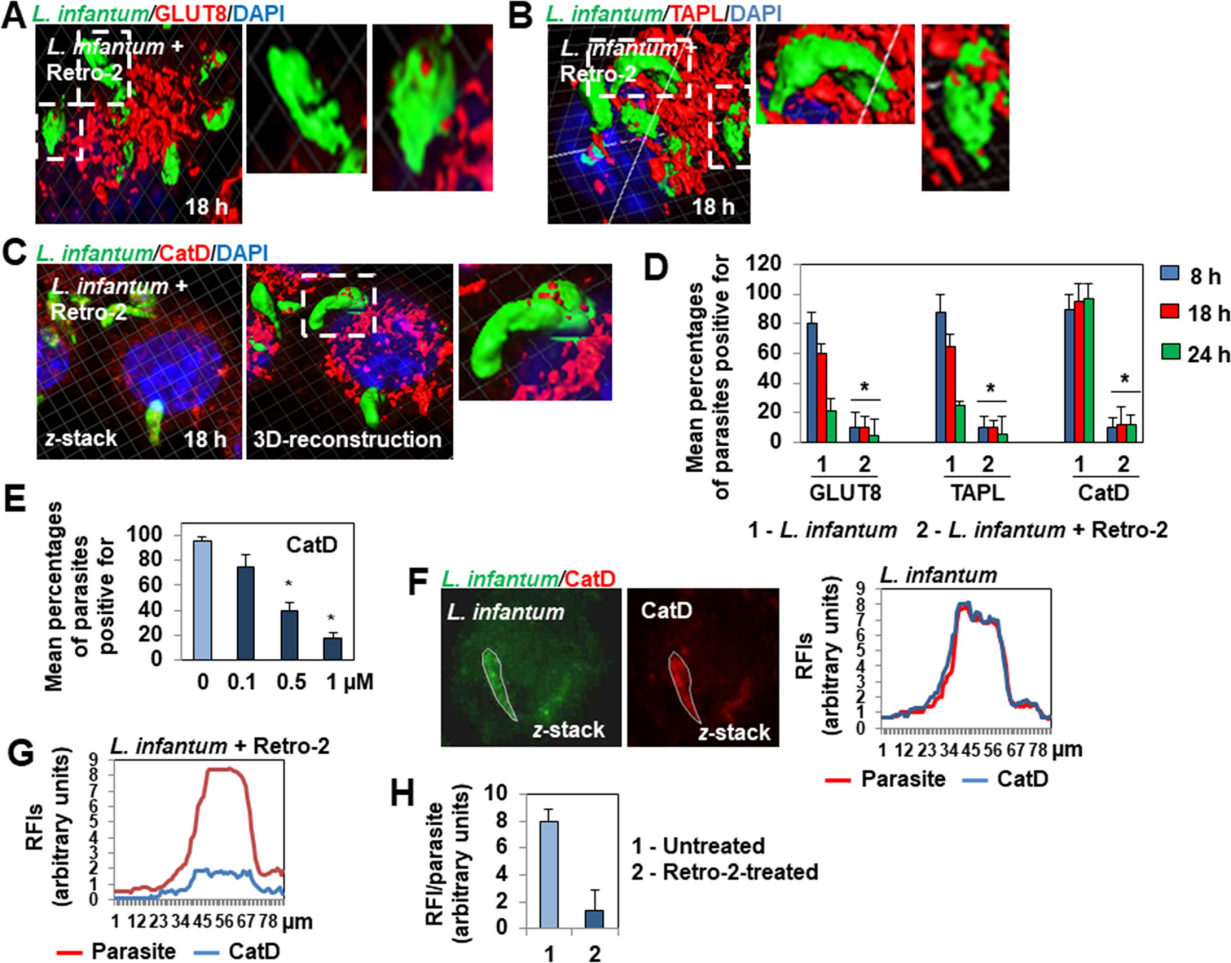
Lack of decoration of *L*. *infantum* LEM 5700 parasites with lysosome-associated hydrolase cathepsin, hexose transporter GLUT8, and polypeptide transporter TAPL in macrophage-like RAW 264.7 cells infected in the continuous presence of Retro-2. **(A)** 3D-reconstruction surface rendering confocal micrograph showing the absence of decoration of fusiform-like and ellipsoidal parasite forms with GLUT8-positive patches in a Retro-2 (1 μM)-treated cell. See Fig 2F for control decoration of parasites in untreated cells. **(B)** 3D-reconstruction surface rendering confocal micrograph showing the absence of decoration of fusiform-like and ellipsoidal parasite forms with TAPL-positive patches in a Retro-2 (1 μM)-treated cell. See Fig 2G for control decoration of parasites in untreated cells. **(C)** *z*-stack and 3D-reconstruction surface rendering micrograph showing the absence of decoration with CatD-positive patches of fusiform-like and ellipsoidal parasite forms hosted in Retro-2 (1 μM)-treated cells. See also S4 Video. See Fig 2H for control decoration of parasites in untreated cells. **(D)** Bar graph showing the decrease in the percentage of parasites decorated with GLUT8-, TAPL- and CatD-positive patches during an infection time-course in cells infected in the continuous presence, or not, of Retro-2 (1 μM). **(E)** Bar graph showing the average percentage of parasites positive for CatD in cells infected in the continuous presence, or not, of increasing concentrations of Retro-2. **(F)** Confocal micrographs (*z*-stack projection) showing green (parasite) and red immunofluorescence labeling (cathepsin D, CatD) in a delineated fusiform-like parasite hosted in an untreated infected cell. Profile showing the scanning analysis of the relative fluorescence intensity (RFI) of green and red fluorescence signals in the delineated fusiform-like parasite. **(G)** A representative profile showing the scanning analysis of RFIs of green and red fluorescence signals measured in a fusiform-like parasite hosted in a Retro-2-treated infected cell. **(H)** Bar graph showing the RFIs of CatD measured in fusiform-like parasites hosted in untreated infected cells and Retro-2 (1 μM)-treated infected cells. Confocal micrographs are representative of three independent experiments. High-magnification images correspond to the white dashed boxes. The percentage of parasites associated with TAPL, GLUT8, or CatD were determined by counting parasites hosted in at least 30 cells at each time-point for each condition. The RFIs were measured by analyzing 8 to 10 parasites for each condition. Data are represented as the average ± SEM. Data are from two independent experiments in duplicate and were analyzed by the unpaired Student t test. *p < 0.01.

**Fig 5:**
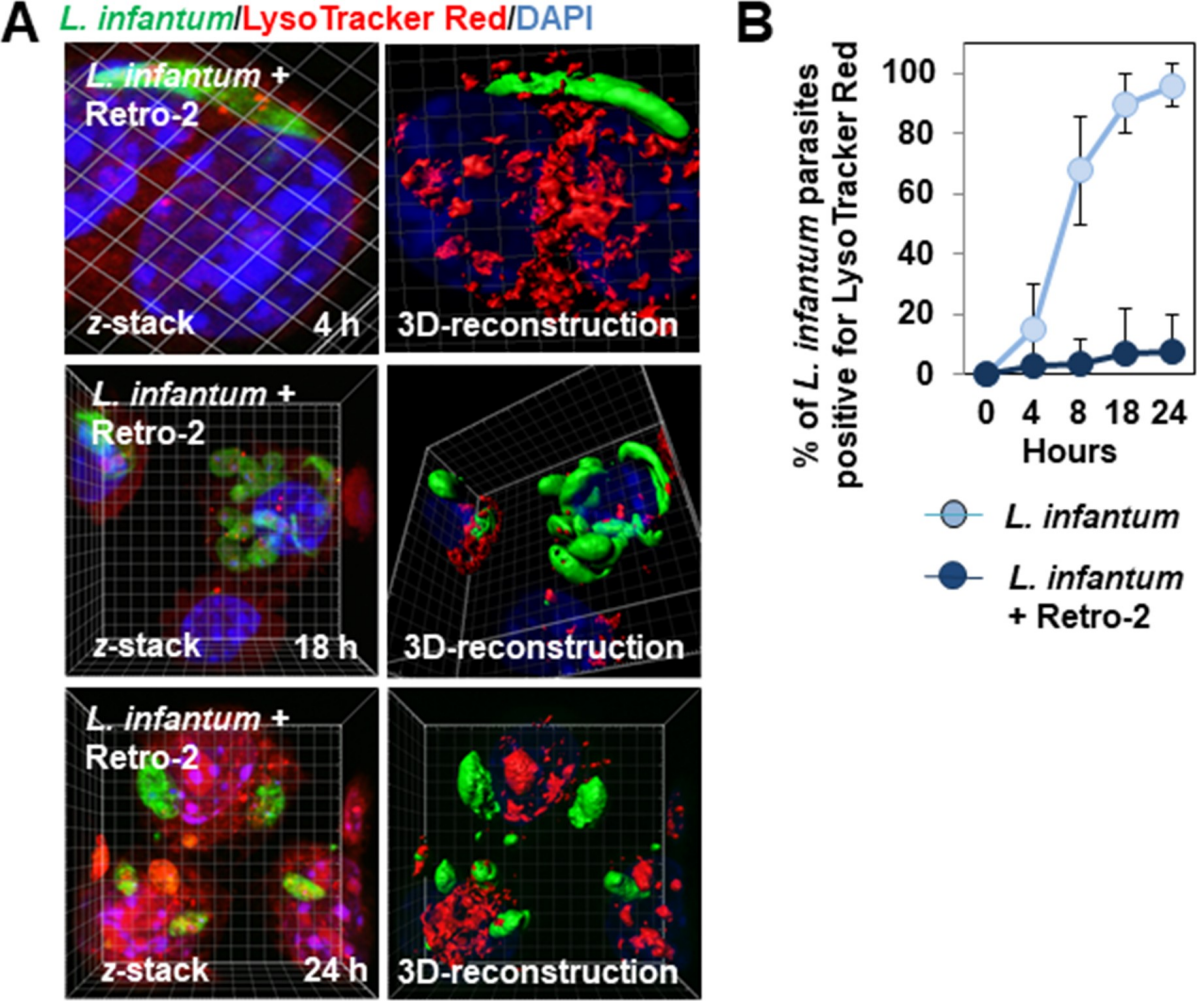
Lack of acquisition of LysoTracker Red positivity by *L*. *infantum* LEM 5700 parasites during an infection time-course in macrophage-like RAW 264.7 cells infected in the continuous presence of Retro-2. **(A)** *z*-stack and 3D-reconstruction surface rendering confocal micrograph showing the absence of acquisition of Lysotracker Red by fusiform-like and ellipsoidal parasites hosted in Retro-2 (1 μM)-treated infected cells during an infection time-course. See Fig 2I for control LysoTracker Red positivity of parasites in untreated cells. **(B)** Graph showing an increase in the percentage of parasites positive for LysoTracker Red in untreated infected cells and the absence of an increase of parasite LysoTracker Red-positivity in Retro-2 (1 μM)-treated infected cells during an infection time-course. Confocal micrographs are representative of two independent experiments. The high-magnification image corresponds to the white dashed box. The percentage of positive parasites was determined by counting parasites hosted in at least 30 cells at each time-point for each condition. Data are presented as the average ± SEM.

3D-reconstruction surface rendering confocal micrographs also showed there to be a marked decrease or the absence of decoration of fusiform-like and ellipsoidal parasite forms by the lysosomal membrane-associated GLUT8 (Fig 4A and 4D) and TAPL (Fig 4B and 4D) transporters in 8 h-, 18 h- and 24 h-infected Retro-2-treated cells relative to untreated infected cells. Consistent with these results, 3D-reconstruction surface rendering confocal micrographs and quantification showed the almost total absence of decoration by the lysosomal luminal-associated CatD hydrolase of fusiform-like and ellipsoidal parasite forms hosted in 8 h-, 18 h- and 24 h-infected Retro-treated cells relative to untreated infected cells (Fig 4C and 4D, and S4 Video). Quantification of the number of intramacrophage parasites decorated by CatD showed that the decrease of parasite decoration developed as a function of the concentration of Retro-2 (Fig 4E). For the CatD-decorated fusiform-like parasites hosted in Retro-2-treated infected cells (Fig 4C), scanning of the CatD RFI in fusiform-like parasites showed the level of decoration per parasite to be dramatically lower than that of fusiform-like parasites hosted in untreated infected cells (Fig 4F to 4H). In addition, LysoTracker Red fluorescence did not enwrap the parasites in cells infected in the presence of Retro-2 (Fig 5A and 5B), contrasting with the extensive enwrapping of parasites hosted in infected untreated cells (Fig 2H). The observed decrease of association with LE- and lysosome-associated molecules was not the consequence of Retro-2-triggered alteration of the cellular presence or distribution of LES and lysosomes, as CLSM images and quantification showed that the cell expression and distribution of Lamp-2- and CadD-positive vesicles were not modified in Retro-2-treated cells relative to that of untreated cells (S4 Fig).

Overall, these results show that Retro-2 impaired the acquisition of host cell LE- and lysosome-associated markers by *L*. *infantum*-containing phagosomes hosted in macrophage-like cells, suggesting that the small trafficking blocker arrested the heterotypic interactions between these vacuolar compartments. To confirm that Retro-2 triggers failure of the heterotypic interaction of donor vacuoles with lysosomes, we conducted a control experiment in a non-infectious cellular autophagy model in which the fusion of donor autophagosomes with recipient lysosomes leads to the formation of the final degradative vacuolar compartment, autolysosomes [34,35]. We used HeLa cells stably expressing the autophagy marker microtubule-associated protein 1 light chain 3 (LC3) coupled to green fluorescent protein (GFP-LC3). As expected [36], nutrient starvation induced the strong presence of autolysosomes in the cell cytoplasm (S5A Fig, Middle image, and S5B Fig) versus their absence in non-starved cells (S5A Fig; left image, and S5B Fig). In contrast, there was an absence of autolysosomes and the strong presence of autophagosomes in nutrient-starved Retro-2-treated cells, showing the failure of fusion between autophagosomes and lysosomes induced by the small compound (S5A Fig, right image, and S5B Fig). We next analyzed autophagic flux by treating the cells with the lysosomotropic agent, chloroquine (CQ), which inhibits lysosomal protease degradation of LC3-II [36]. Western blotting showed the abundance of LC3-II protein to be no higher in nutrient-starved Retro-2-treated cells in the presence of CQ than nutrient-starved Retro-2-treated cells without CQ (S5C Fig), showing that Retro-2 blocks autophagic flux.

### Permanent residence of *L*. *infantum* LEM 5700 parasites in immature phagosomes negatively affects parasite differentiation and survival

**Fig 6:**
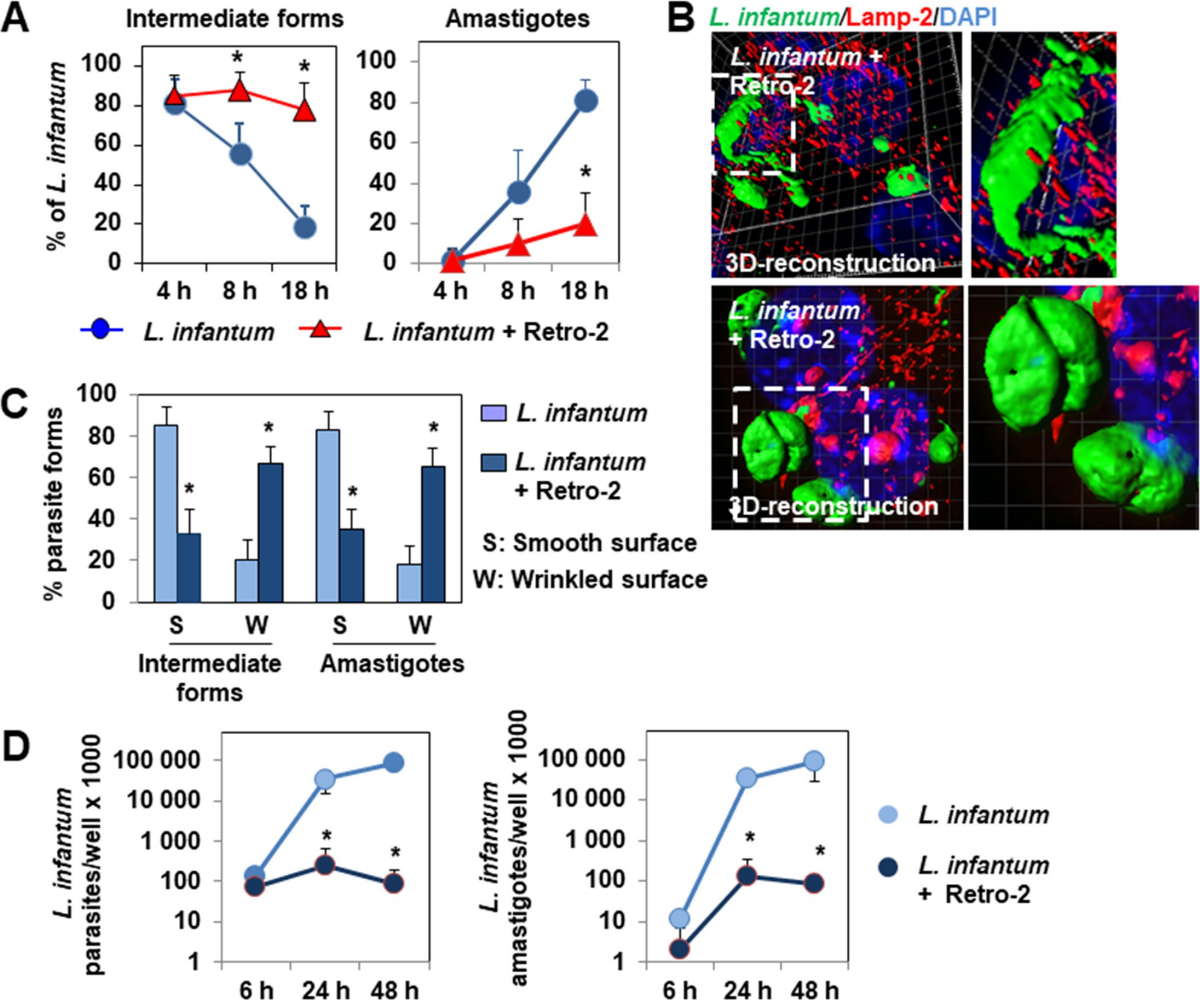
*L*. *infantum* LEM 5700 parasites are morphologically altered and die when hosted in Retro-2-treated macrophage-like RAW 264.7 cells. **(A)** Graph showing the percentage of intermediate parasite forms and amastigotes during an infection time-course in cells infected in the continuous presence, or not, of Retro-2 (1 μM). **(B)** 3D-reconstruction surface rendering confocal micrographs showing representative altered fusiform-like and ellipsoidal forms of parasites with a wrinkled cell surface, forming deep undulations when hosted in 18 h-Retro-2-treated infected cells. For comparison, see the smooth surface of parasite forms hosted in untreated infected cells in Fig 1. **(C)** Bar graph showing the percentage of intermediate parasite forms and amastigotes exhibiting a smooth or wrinkled body surface in cells infected for 18 h in the continuous presence, or not, of Retro-2. **(D)** Graph showing the parasite (left) and amastigote (right) loads determined by RT-qPCR in cells infected in the continuous presence, or not, of Retro-2 during an infection time-course. Confocal micrographs are representative of two independent experiments. The percentage of intermediate parasite forms and amastigotes was determined by analyzing at least 40 cells for each time-point for each condition. The percentage of parasites with a smooth or wrinkled surface was determined by analyzing parasites hosted in at least 30 cells for each time-point for each condition. Parasite and amastigote levels per culture well were quantified by RT-qPCR in two independent experiments in duplicate. Data are presented as averages ± SEM and were analyzed using the unpaired Student t test. *p < 0.01.

We next examined whether impairment of the heterotypic interaction between the *L*. *infantum*-containing phagosomes and the macrophage LEs and lysosomes has an impact on parasite differentiation. Quantitative imaging showed Retro-2-treated infected cells to contain mostly intermediate forms that showed a marked delay in transformation into amastigotes (Fig 6A). This observed delay of differentiation of intramacrophage parasites was not simply a direct effect of the compound on the parasites, as the differentiation of Retro-2-treated axenic parasites was not altered (S6 Fig). CLSM analysis showed a high number of *L*. *infantum* intermediate and amastigote forms residing in Retro-2-treated infected cells with a highly-altered cell surface (Fig 6B and 6C), which strongly differed from that of the regular body surface of parasites hosted in untreated infected cells (Fig 1). RT-qPCR quantification showed marked time-dependent lowering of the parasite and amastigote burden in Retro-2-treated infected cells (Fig 6D). Overall, these results provide evidence that the maturation of *L*. *infantum*-containing phagosomes is necessary for *L*. *infantum* parasites to successfully complete promastigote-to-amastigote differentiation and survive.

### Routing of *L*. *infantum* LEM 5700-containing phagosomes toward LEs and lysosomes is microtubule (MT) dependent

**Fig 7:**
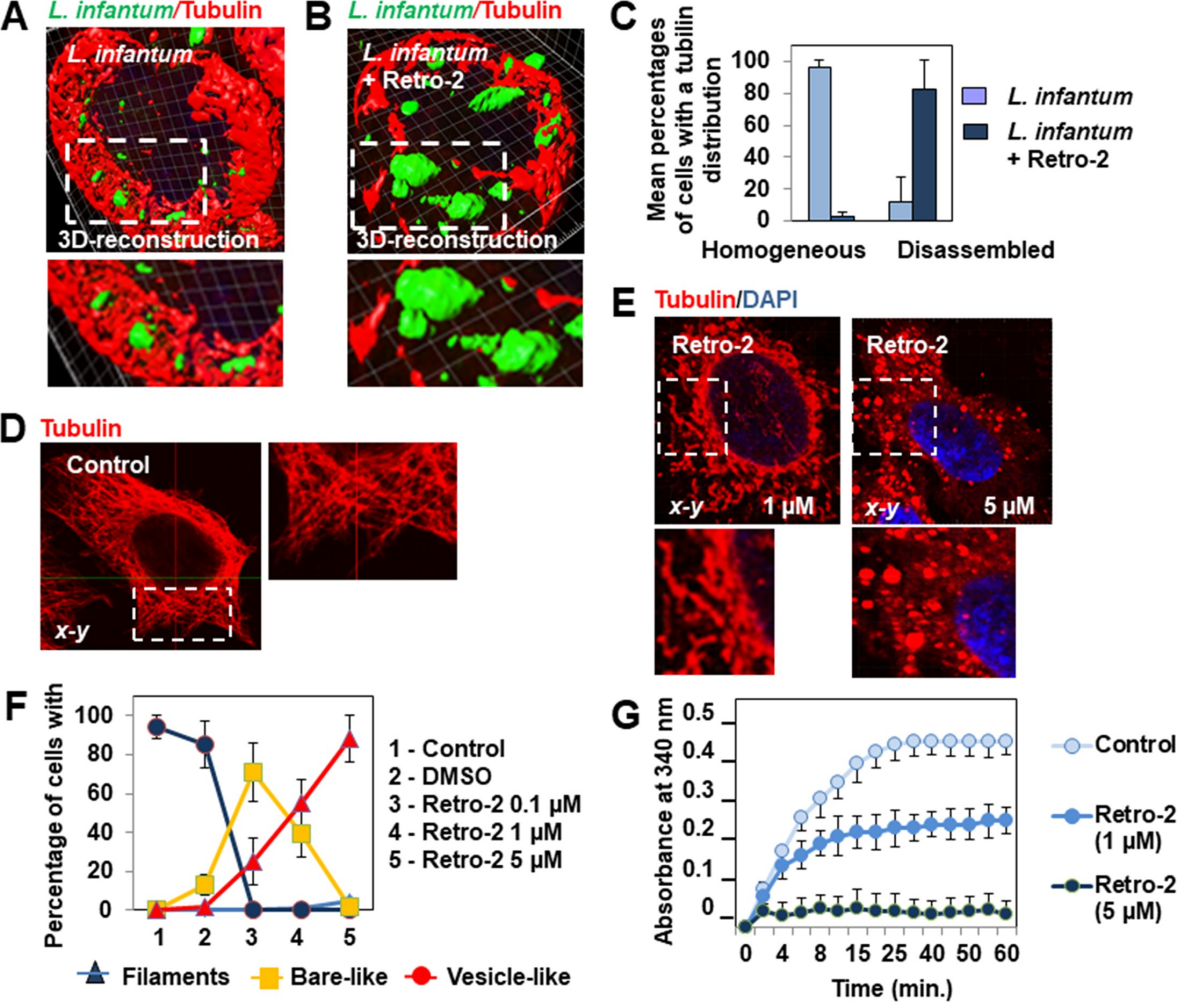
Retro-2 treatment results in disassembly the cell microtubule network. **(A)** 3D-reconstruction surface rendering confocal micrograph showing the well-ordered perinuclear distribution of tubulin immunolabeling in an untreated infected macrophage-like cell. **(B)** 3D-reconstruction surface rendering confocal micrograph showing the dispersion of tubulin immunolabeling in a cell infected for 24 h in the continuous presence of Retro-2 (1 μM). **(C)** Bar graph showing the percentage of macrophage-like cells infected in the continuous presence, or not, of Retro-2, with homogeneous perinuclear or dispersed tubulin immunolabeling. **(D)** *x-y* confocal micrograph showing the MT network in an untreated epithelial HeLa cell. (**E**) *x-y* confocal micrograph showing marked disorganization of the MT network in 24 h-Retro-2-treated HeLa cells. Note the appearance of short tubulin-positive bar-like (1 μM) and vesicle/aggregate (5 μM) structures distributed throughout the cell cytoplasm. (**F**) Graph showing quantification of cells expressing well-organized tubulin-positive fibers, dispersed bar-like structures, or vesicle-like aggregates in untreated cells and cells treated for 24 h with DMSO or increasing concentrations of Retro-2. (**G**) Graph showing the effect of increasing concentrations of Retro-2 on GTP-dependent tubulin polymerization *in vitro*. Confocal micrographs are representative of two independent experiments in duplicate. High-magnification images correspond to the white dashed boxes. Tubulin in the reaction buffer was incubated at 37°C in the presence of vehicle (DMSO) or Retro-2 and polymerization measured by spectrophotometry. Data were obtained from two independent experiments. Data are presented as the mean ± SEM.

The functional maturation process of phagosomes implies dynein-dependent movements of early phagosomes along MT tracts followed by their fusion with lysosomes controlled by SNAREs-containing membrane fusion platforms[6]. The above observed lack of decoration of poorly differentiated *L*. *infantum* parasites by LE and lysosomal markers in Retro-2-treated infected macrophage-like cells may result from the absence of *L*. *infantum*-containing phagosome movement along MT tracks or downstream impairment of SNAREs-dependent membrane fusion with LEs and lysosomes. Thus, we first examined the organization of the MT network in Retro-2-treated infected macrophage-like cells. Unexpectedly, 3D-reconstruction surface rendering confocal micrographs revealed the dense tubulin network in *L*. *infantum* LEM 5700-infected macrophage-like cells (Fig 7A and 7C) to be highly disorganized in cells infected for 24 h in the continuous presence of Retro-2 (Fig 7B and 7C). In these cells, large spaces devoid of tubulin and dispersed fragmented tubulin-positive bar-like structures were evident. We validated this unexpected observation by conducting a control experiment with epithelial HeLa cells in which the MT network consisted of straight MT filaments well-oriented from the cell nucleus toward the membrane (Fig 7D). CLMS analysis showed that, as in macrophage-like cells, 24 h of treatment with Retro-2 triggered dramatic concentration-dependent disorganization of the HeLa MT network, characterized by the concentration-dependent appearance of dispersed tubulin-positive bar-like and vesicle/aggregate structures (Fig 7E and 7F). We finally confirmed the ability of Retro-2 to induce concentration-dependent MT disassembly using an *in vitro* tubulin polymerization assay (Fig 7G).

### Nocodazole and siRNA *dynein* silencing mimic the effects of Retro-2

**Fig 8:**
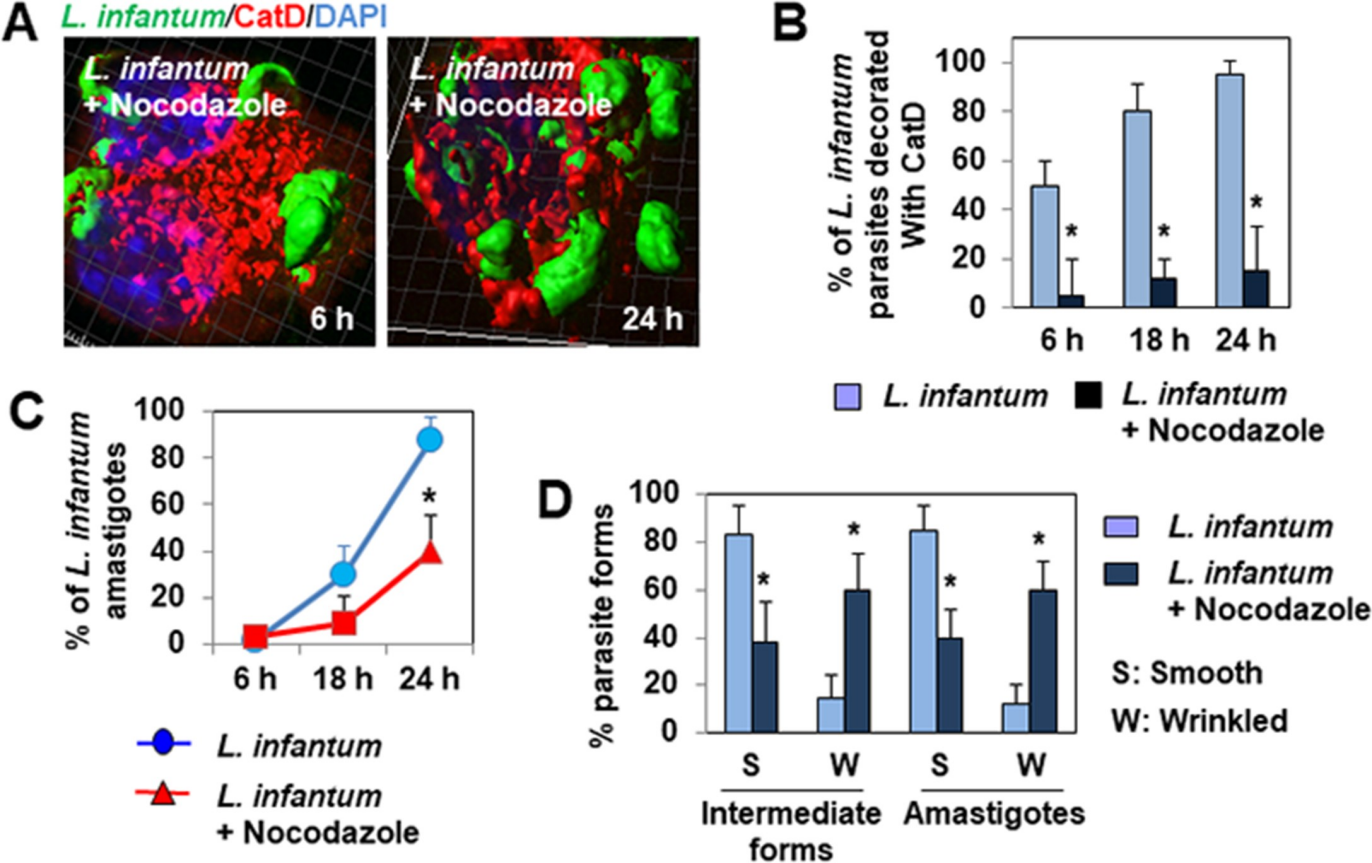
The microtubule-disassembly agent nocodazole abolishes the maturation of *L*. *infantum* LEM 5700-containing phagosomes, delays parasite differentiation, and promotes parasite death in macrophage-like Raw 264.7 cells. **(A)** 3D-reconstruction surface rendering confocal micrographs showing limited association of cathepsin D (CatD) with parasites hosted in nocodazole-treated infected cells. For comparison, see the CatD decoration of parasites hosted in untreated cells in Fig 1G. **(B)** Bar graph showing the percentage of parasites decorated with CatD in cells infected in the continuous presence, or not, of nocodazole during a time-course of infection. **(C)** Graph showing the time-dependent evolution of the percentage of amastigotes in cells infected in the continuous presence, or not, of nocodazole (1 μM) during a time-course of infection. **(D)** Bar graph showing the percentage of intermediate parasite forms and amastigotes exhibiting a smooth or wrinkled body surface when hosted in cells infected in the continuous presence, or not, of nocodazole. Confocal micrographs are representative of two independent experiments. The percentage of parasites was determined by analyzing at least 30 cells for each time-point for each condition. Data are presented as averages ± SEM and were analyzed using the unpaired Student t test. *p < 0.01.

We confirmed the pivotal role of MTs in the maturation process of *L*. *infantum* LEM 5700-containing phagosomes/PVs by infecting macrophage-like cells in the continuous presence of the reference MT-disassembling agent (MDA), nocodazole [37]. 3D-reconstruction surface rendering analysis showed the elevated presence of hosted fusiform-like and ellipsoidal parasites in nocodazole-treated 24 h-infected cells (Fig 8A). The maturation of *L*. *infantum*-containing phagosomes/PVs hosted in macrophage-like cells infected in the continuous presence of nocodazole was impaired, shown by the scarcity of CatD decorating the hosted parasites (Fig 8A and 8B). The continuous treatment of cells with nocodazole impaired parasite differentiation, shown by the prolonged elevated presence of the intermediate forms of the parasite, with the concomitant delayed appearance of amastigotes (Fig 8C), as in Retro-2-treated infected cells (Fig 6A). Moreover, the parasites hosted in nocodazole-treated infected cells had an altered cell surface (Fig 8A and 8D), such as those in infected cells treated with Retro-2 (Fig 6B and 6C).

**Fig 9:**
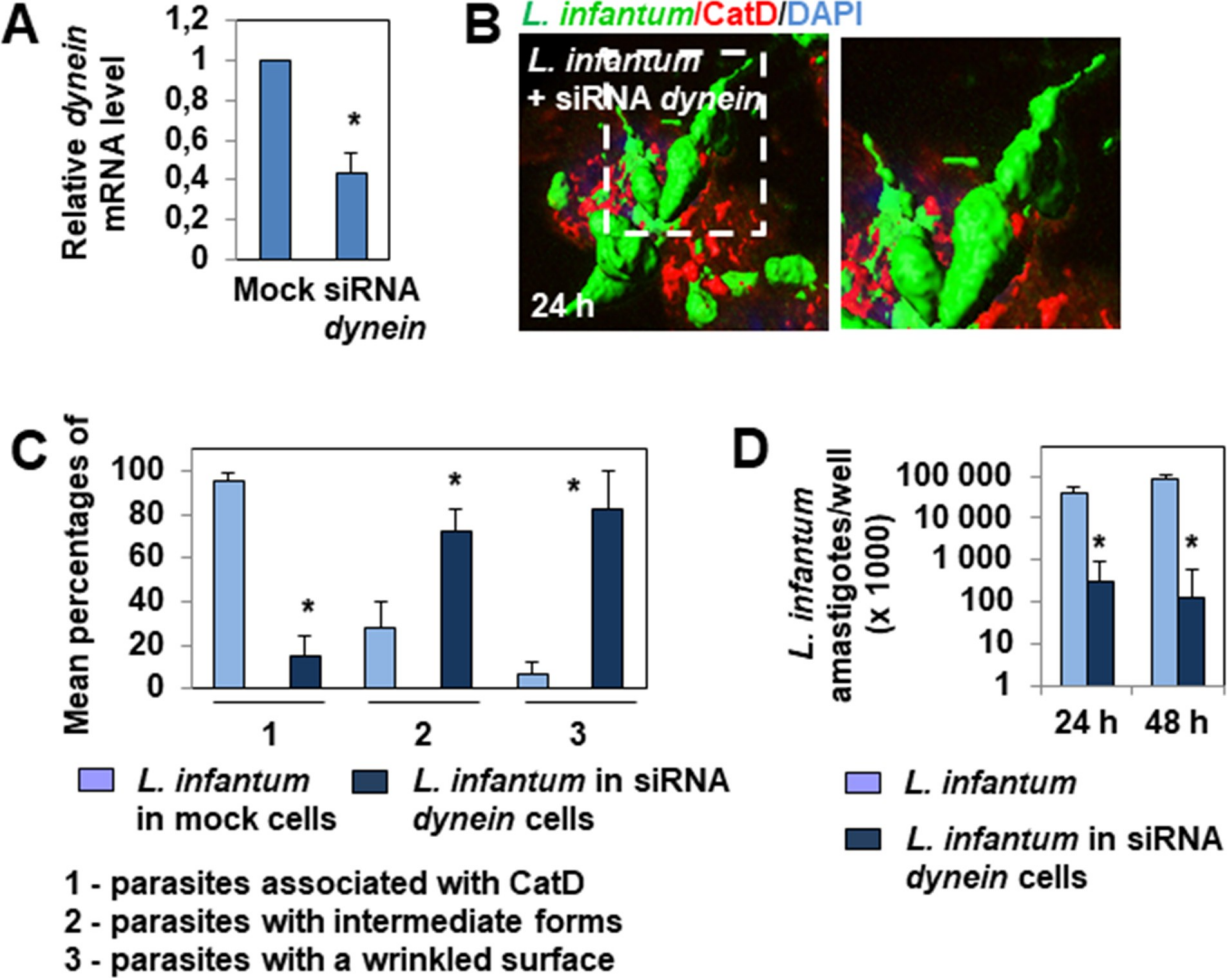
Silencing of the *dynein* gene in macrophage-like Raw 264.7 cells abolishes the maturation of *L*. *infantum* LEM 5700-containing phagosomes/PVs, delays parasite differentiation, and promotes parasite death. **(A)** Bar graph showing *dynein* mRNA levels relative to those of *gapdh* mRNA measured by RT-qPCR in mock and *dynein* siRNA-transfected cells. **(B)** 3D-reconstruction surface rendering confocal micrograph showing limited association of cathepsin D (CatD) with fusiform-like parasites hosted in an infected dynein siRNA-transfected cell, and the highly altered morphology of these parasites. For comparison, see the CatD decoration of parasites hosted in untreated cells in Fig 1G. **(C)** Bar graph showing the percentage of parasites associated with CatD, the percentage of intermediate parasite forms and amastigotes, and the percentage of parasites with a wrinkled cell surface in mock-transfected and siRNA *dynein*-transfected cells. **(D)** Bar graph showing the amastigote load determined by RT-qPCR in mock-infected cells and *dynein* siRNA-transfected infected cells. The confocal micrograph is representative of two or three independent experiments. The high-magnification view corresponds to the white dashed box. The percentage of parasites was determined by analyzing at least 30 cells for each condition. The percentage of parasites with a smooth or wrinkled cell surface was determined by analyzing parasites hosted in at least 20 cells for each condition. *Dynein* mRNA levels were quantified by RT-qPCR of over three independent biological samples for each time-point. Amastigote levels per culture well were quantified by RT-qPCR in two independent experiments in duplicate. Data are presented as averages ± SEM and were analyzed by the unpaired Student t test. *p < 0.01.

We completed our analysis by silencing the cellular gene *dynein*. This gene codes for the MT-associated motor minus-end-directed dynein [38], which generates rapid unidirectional movement of phagosomes towards lysosomes along MTs [39]. RT-qPCR showed siRNA-mediated silencing of the *dynein* gene to be effective in macrophage-like RAW 264.7 cells (Fig 9A). The siRNA *dynein*-transfected infected cells showed a dramatic reduction in the maturation of the *L*. *infantum* LEM 5700-containing phagosomes/PVs and a delay in the differentiation of the insulated parasites (Fig 9B and 9C), as observed in Retro-2-treated infected cells (Figs 2 and 3, and Fig 6A) and nocodazole-treated infected cells (Fig 8A to 8C). Moreover, the fusiform-like parasites hosted in siRNA *dynein*-transfected infected cells expressed a strong altered surface (Fig 9B and 9C), similar to that of the parasites hosted in Retro-2-treated (Fig 6B and 6C) and nocodazole-treated (Fig 8D and 8E) infected cells. In addition, RT-qPCR quantification showed that the parasites in siRNA *dynein*-transfected infected cells died, shown by marked lowering of the amastigote burden (Fig 9D), as found in infected cells treated with Retro-2 (Fig 6D).

## Discussion

**Fig 10:**
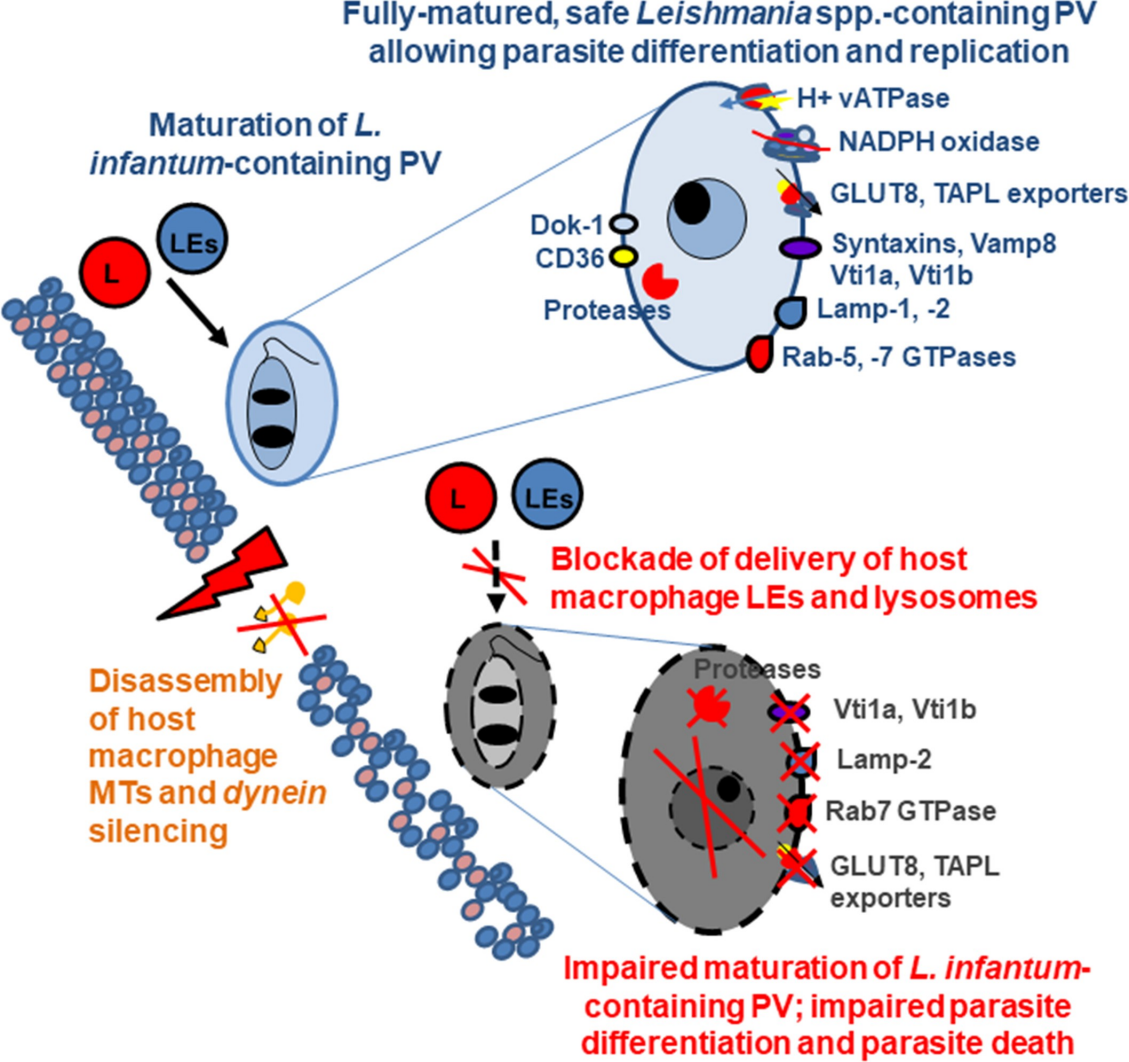
Scheme summarizing the abolition of maturation of tight-fitting *L*. *infantum*-containing PVs by structurally and functionally altering the MT network of macrophage-like cells. Such alteration of the MT network results in most of the phagocytosed *L*. *infantum* promastigotes remaining permanently insulated in non-mature phagosomes, in which they do not undergo normal promastigote-to-amastigote differentiation and finally die.

Our work provides evidence that the maturation of *L*. *infantum*-containing phagosomes in infected macrophages is dependent on microtubule dynamics and highlights the pivotal role of the fusion of parasite-containing phagosomes to the host cell endolysosomal vacuolar compartments to allow the full promastigote-to-amastigote differentiation crucial for parasite survival and development. Our results add to existing knowledge on the maturation process of *L*. *infantum*-containing phagosomes/PVs by newly examining the time-dependent appearance/disappearance of two membrane-associated SNAREs, Vti1a and Vti1b, and two membrane-associated lysosomal transporters, GLUT8 and TAPL (Fig 10) in comparison with the appearance/disappearance of the classical *bona fide* endolysosomal markers Rab7, Lamp-2, and CatD, necessary for the morphological differentiation of *L*. *infantum* insulated in tight-fitting PVs hosted in macrophage-like cells. Vacuolar fusion with lysosomes occurs by two processes, the first consisting of a continuous cycle of transient membrane contacts that mainly allow the transfer of the lysosomal luminal content and the second, by full membrane fusion, allowing both the transfer of lysosomal membrane-associated proteins and lipids and luminal content [40]. Desjardins [41] proposed that phagolysosome biogenesis involves heterotypic fusion with host-cell lysosomes by a kiss-and-run mechanism of fusion. The observed acquisition of lysosomal membrane-associated GLUT8 and TAPL by *L*. *infantum*-containing PVs, together with the previously observed presence of the lysosomal membrane-associated vATPase in *L*. *donovani*-containing PVs [9,10], suggests that a certain amount of lysosomal membrane is probably acquired during membrane fusion. Early acquisition of the lysosomal membrane GLUT8 and TAPL transporters by the *L*. *infantum*-containing PVs, followed by their disappearance, is surprising but may be due to the fact that they may be deleterious for the intravacuolar lifestyle of the parasite. Indeed, these transporters function in lysosomes as exporters for the recycling of degradation products [42]. Thus, they may deplete the mature PVs of nutrients useful for parasite growth. Whether *Leishmania* excludes these lysosomal exporters by the same mechanism that leads to the exclusion of the vATPase from the PV membrane [10] is yet to be demonstrated.

We show that the maturation of *L*. *infantum*-containing phagosomes in tight-fitting PVs is MT- and dynein-dependent (Fig 10), but the precise nature of the interaction of the *L*. *infantum*-containing phagosomes with the MT-associated dynein remains unclear. It is possible that it interacts with dynein similarly to phagosomes, for which fusion with lysosomes involves the membrane-bound Rab7 GTPase. In this case, following clustering within phagosome membrane microdomains, Rab7 GTPase recruits the MT-associated dynein-dynactin complex that powers minus-end transport along MTs [39,43–45]. As we found here for *L*. *infantum*, Rab7 is present at the membrane of tight-fitting PVs that insulate *L*. *major* and *L*. *donovani* [9,27,32,46]. This GTPase appears to play an important role in the maturation of *L*. *donovani*-containing phagosomes, as they become low-fusogenic when it is missing [32]. It has been elegantly demonstrated that the lipophosphoglycan (LPG) of *L*. *donovani* inhibits the motion of parasite-containing phagosomes by altering the membrane-associated microdomains [7,8,10,11,32,47]. Our observation of a MT- and dynein-dependent mechanism controlling the maturation process of *L*. *infantum*-containing phagosomes could explain how LPG delays the fusion of *L*. *donovani*-containing phagosomes with LEs and lysosomes [7,47]. It is possible that LPG arrests the movement of *L*. *donovani*-containing early phagosomes by affecting the known positioning of the dynein motor within the cell membrane microdomains [39], resulting in its disconnection from with the PV membrane-associated Rab7.

Insulated within infected host mammalian cells, *Leishmania* promastigotes encounter various stressful conditions and environmental changes [48,49] and undergo differentiation, characterized by large morphological and functional changes. The final transformation into amastigotes is crucial, since this form of the parasite is particularly adapted to live within the acidic, hydrolytic, and nutrient-poor environment and resist attacks by the defensive mechanisms of the macrophage [50]. Moreover, metabolic adaptation of the parasite is particularly important, as it includes the downregulation of many surface nutrient transporters and changes in central carbon metabolism [51]. We show that permanent inhibition of the interaction of the *L*. *infantum*-containing phagosomes/PVs with host cell LEs and lysosomes delays their maturation, resulting in immature phagosomes/PVs (Fig 10). This situation is extremely deleterious for the insulated parasites, as they do not complete promastigote-to-amastigote differentiation and die. Microbial pathogens and parasites insulated in intracellular vacuoles have developed specific sophisticated strategies to import host-cell cytosol nutrients into the lumen of the pathogen-containing phagosomes/PVs to support their intracellular growth [52]. Insulated *Leishmania spp*. require a diverse range of host energy sources, including sugars, amino acids, and lipids to survive intracellularly [53]. The acquisition of host-cell nutrients likely occurs through the constant fusion of *Leishmania spp*.-containing PVs with the host cell lysosomes containing degraded cargo [27,28,31,46], thus providing the carbon sources necessary for growth of the parasite [54,55]. We hypothesize that the observed death of the *L*. *infantum* parasite occurs by nutrient starvation. This is based on our observation that pharmacologically induced failure of the MT-dependent fusion between *L*. *infantum*-containing phagosomes and lysosomes results in their accumulation in a permanently immature state. This leads to an impoverished nutritional status because of the lack of acquisition of degraded lysosomal products. Further experiments are needed to demonstrate the scarcity of nutrients in the permanently immature phagosomes/PVs, although it is recognized that the direct measurement of nutrient levels in this vacuolar compartment is difficult [54].

The cellular vacuolar trafficking of several toxins, bacterial pathogens, and viruses is negatively affected by the small inhibitor Retro-2 [56]. Knowledge of the regulatory mechanisms that control such trafficking suggests that the inhibitory effects of Retro-2 are the result of its actions on various targets. Stx5-dependent effects [29,57,58] relate to a Retro-2-induced default of Stx5 localization to the TGN because it impairs Sec16A-dependent cycling of this SNAREs between the Golgi and the ER [30] and inhibits the ASNA1-mediated ER targeting and insertion of tail-anchored proteins [59]. Moreover, it promoted a default of the docking of EEs carrying Shiga toxin onto the TGN because the EE-associated Shiga toxin-trafficking chaperone GPP130 can no longer bind to its TGN-associated Stx5 receptor [30]. However, effects of Retro-2 at sites other than the TGN-ER have been reported, such as the blocking of cell entry of Herpes Simplex virus [60] and filovuruses [61], and the decrease of the size of the very large, communal PV insulating *L*. *amazonensis* which is formed by fusion with LEs and lysosomes [33]. The observation that Retro-2 disassembles the cellular MT network raises certain questions. Indeed, this network is required for endogenous vacuolar trafficking essential for cellular homeostasis [62,63]. As a consequence, Retro-2 would be expected to have negative pleiotropic effects and yet, this is not the case [29]. Identical questions have been raised [64] concerning the blocking effect of Retro-2 at the ER [30,59]. For the effect on MT network it is possible that Retro-2 selectively affects one or several existing stable and dynamic sub-populations of αβ-tubulin dimers, or stable MTs with numerous post-translational modifications of tubulin, or various cellular pools of MTs [65,66]. Future studies are required to resolve this issue.

Our study and others [33,67,68] demonstrate that *Leishmania spp*.-containing PVs constitute a druggable target for the treatment of leishmaniasis [69,70]. This is especially important in the context of the current therapeutic arsenal that is rapidly becoming ineffective due to the accelerated propagation of drug resistance in *Leishmania spp*. [71].

## Materials and methods

### Reagents, siRNAs and antibodies

Retro-2 **(**2,3-Dihydro-2-(5-methyl-2-thienyl)-3-phenyl-4(1H)-quinazolinone), chloroquine, nocodazole, and rabbit anti-actin (A2066) were purchased from Sigma-Aldrich **(**Saint Quentin Fallavier, 38070 France). LysoTracker Red and 4′,6-diamidino-2-phenylindole (DAPI) were purchased from Invitrogen (Life Technologies, ThermoFisher Scientific, Courtaboeuf, 91941 France). RT-qPCR primers (forward and reverse) and siRNA dynein were purchased from Eurogentec (Angers, France). The TransIT-X2 Dynamic Delivery System was purchased from Euromedex (Souffelweyersheim, France). Mouse mAb 2A3-26 directed against a plasma membrane antigen of *L*. *amazonensis* amastigotes [72] and recognizing *L*. *infantum* (this study) was kindly donated by Dr E. Prina (Institut Pasteur, Paris, France). Mouse anti-cathepsin D mAb (E7, sc-13148), anti-Stx5 (B-8), and rabbit anti-Rab7 (sc-10767) were purchased from Santa Cruz Biotechnology Inc. Rabbit anti-Lamp-2/CD107b was purchased from Thermo Fisher Scientific (Villebon sur Yvette, 91963 France). Mouse anti-Vti1a (D8U3M) and anti-α-tubulin mAbs (11H10) were purchased from Cell Signaling Technology (Ozyme, Saint Quentin en Yvelines, 78053 France). Mouse anti-Vti1b mAb (clone 7) was purchased from BD Biosciences (Le Pont de Claix, 38801 France). Rabbit anti-GLUT8 [73] was kindly provided by Pr. A Schürman (German Institute of Human Nutrition Potsdam-Rehbrücke, Nuthetal, Germany). Rabbit anti-TAPL [74] was kindly provided by Pr. R. Abele (German Institute of Human Nutrition Potsdam-Rehbrücke, Nuthetal, Germany). Alexa-488 and -594-conjugated anti-rabbit and anti-mouse secondary antibodies were purchased from Interchim (Montluçon, 03100, France) and Euromedex. Anti-LC3 antibody (L7543), anti-β-actin-peroxidase (A3853), goat anti-rabbit IgG- and sheep anti-mouse IgG-peroxidase antibodies (A0545 and A5906, respectively) were purchased from Sigma-Aldrich.

### *L*. *infantum* LEM 5700 culture

*L*. *infantum* strain LEM 5700 (MHOM/FR/2008/LEM5700—zymodeme MON-1) was provided by the Centre National de Ressources des *Leishmania* (CRB-Leish) (Université de Montpellier, Centre Hospitalier Universitaire (CHU), UMR MIVEGEC CNRS 5288/IRD 224/UM, Département de Parasitologie—Mycologie, 34295 Montpellier, France). This strain was isolated in the Department of Pediatrics of the CHU Montpellier from the bone marrow of an eight-year-old girl suffering from visceral leishmaniasis. Parasites were grown as previously described in M-199 medium supplemented with 40 mM HEPES, 100 mM adenosine, 0.5 mg/l hemin, and 10% heat-inactivated fetal bovine serum (FBS) at 26°C in a dark environment under an atmosphere of 5% CO_2_ [75]. All cell experiments were performed with *L*. *infantum* promastigotes, the physiologically relevant virulent stage of *Leishmania*, in their stationary phase of growth.

Differentiation of axenic *L*. *infantum* promastigotes into axenic *L*. *infantum* amastigotes was achieved by dilution of 1 x 10^6^ promastigotes in 5 mL axenic amastigote media (M-199 media supplemented with 15 mM KCl, 8 mM glucose, 5 mM glutamine, 2.5% BBL trypticase peptone, 4 mM hemin, and 20% FCS) (pH 5.5) [75].

### Cell culture and infection and treatment protocols

The mouse monocyte/macrophage-like cell line RAW 264.7 (Cell collection CNRS UMR 8076 BioCis, University Paris-Saclay) [75] was cultured in Dulbecco’s modified Eagle's minimal essential medium (DMEM) (Invitrogen Life Technologies) supplemented with 10% heat-inactivated fetal bovine serum, at 37°C in a humidified atmosphere containing 5% CO_2_. For infection, RAW 264.7 cells were placed in 24-well TPP tissue culture plates (ATGC, Marne la Vallée, France), with or without sterile coverslips, and infected with unopsonized, *L*. *infantum* LEM 5700 promastigotes (MOI of 10 parasites per cell) at 37°C in an atmosphere containing 5% CO_2_. For treatments, cells were infected for the indicated time-periods in the continuous presence, or not, of compounds. At the indicated time-periods post-infection, samples were washed three times with cold EBSS to remove free parasites and treated for immunofluorescence labeling. Observation and image acquisition of fixed macrophage-like cultures by confocal microscopy were performed at various times after infection, ranging from 4 to 24 h, depending on the experiment.

HeLa cells and HeLa cells stably transfected with rat GFP-LC3 (GFP-LC3-expressing HeLa cells) were kindly provided by A.M. Tolkovsky (Department of Biochemistry, University of Cambridge, UK) [76]. Cells were seeded and grown in culture plates (TPP, ATGC Biotechnologie, Noisy Le Grand, France) containing coverslips and cultured in RPMI-1640 with L-glutamine (Life Technologies, Cergy, France) at 37°C in an atmosphere containing 5% CO_2_. For autophagy induction by amino-acid deprivation, the cells were incubated for the indicated times in Earle’s balanced salt solution (EBSS) (Sigma) at 37°C in an atmosphere containing 5% CO_2_ [36].

### RNA interference

Transfection of macrophage-like RAW 264.7 cells with siRNA targeting the *dynein* gene (forward [5’-CUGUGAUUGAUGCAGACAAUU-3’] and reverse: [5’-UUGUCUGCAUCAAUCACAGUU-3’]) was performed using the TransIT-X2 Dynamic Delivery System (Euromedex) following the manufacturer’s protocol. Cells were transfected 48 h before infection and collected for experiments.

### Immunofluorescence labelling

*L*. *infantum* LEM 5700 and components of *L*. *infantum* LEM 5700-containing PVs were identified by indirect immunofluorescence labelling. Specimens were fixed with 3% paraformaldehyde in phosphate-buffered saline (PBS) for 5 min at room temperature, washed three times with PBS, treated with PBS containing 50 mM NH_4_Cl for 10 min to neutralize the aldehyde, and blocked by adding PBS containing 0.2% gelatin. Cells were permeabilized by incubation in 0.2% Triton X-100 in PBS for 4 min at room temperature and then washed three times with PBS. Intramacrophage *L*. *infantum* promastigotes and amastigotes were immunolabeled with mAb 2A3-26 (1/500). Immunolabeling of endolysosomal pathway markers was performed using anti-Rab7 (1/200), anti-Lamp-2 (1/200), anti-cathepsin D (1/100), anti-GLUT8 (1/100), anti-TAPL (1/100), anti-Vti1a (1/100), anti-Vti1b (1/100), and anti-Stx5 (1/200) antibodies. Microtubules and the actin cytoskeleton were immunolabeled with anti-α-tubulin (1/500) and anti-actin (1/100) antibodies, respectively. After immunolabeling with primary antibodies, the specimens were developed using appropriate conjugated secondary antibodies (Jackson Immunoresearch). Cells were also stained for 15 min with DAPI (100 μg/ml) (Invitrogen Life Technologies) to visualize the nucleus. Coverslips were mounted using Dako fluorescent mounting medium (Invitrogen Life Technologies) for CLSM examination.

### LysoTracker Red labeling

Cells were labeled with LysoTracker Red (50 nM) by incubating them with the probe for 60 min. The labeled cells were washed three times with sterile PBS, and then fixed with 3% paraformaldehyde in PBS for 5 min at room temperature. Coverslips were mounted using Dako fluorescent mounting medium.

### Confocal laser scanning microscopy (CLSM)

The samples were imaged with an inverted STED-gated Leica TCS SP8 microscope (Leica, Germany) using a HC PL APO CS2 63x/1.40 oil immersion objective lens. The instrument was equipped with a 405-nm diode for DAPI excitation and a WLL Laser. Blue, green, and red fluorescence emission was collected using 410–460, 505–550, and 560–760 nm wide emission slits in sequential mode, respectively. The pinhole was set to 1.0 Airy unit, giving an optical slice thickness of 0.89 μm. Twelve-bit numerical confocal micrographs were processed using Leica SP8 LAS X software (Version 2.0.1; Leica, Germany). Confocal optical sectioning was performed each 0.3 μm along the *z* axis.

### Multidimensional imaging analysis and quantification

Three-dimensional volume renders (3D-reconstruction surface rendering) from confocal microscopy optical sections (*z*-stack) were obtained using Imaris Measurement Pro software (Bitplane AG, Zurich, Switzerland). Isosurface rendering was used to measure the volume of identified structures. To measure the volume of a defined object, confocal *z*-stack images (row data following the Nyquist sampling theorem) were used to perform 3D reconstruction views. The resulting volume rendering data sets were then processed to obtain a surface rendering calculation based on voxel intensities. A smoothing Gaussian filter of 0.09 was applied before thresholding. Imaris provides volumes and surfaces of the structure. Each channel was analyzed separately. Relative fluorescence intensities (RFIs) were determined on maximum-intensity projections using *z*-stacks. The level of a given host cell marker associated with an intramacrophage *L*. *infantum* parasite was quantified using the "segmented line" tool was to cleanly delineate the area relative to the immunolabeled parasite in the *z*-stack micrographs. Once the area of interest was determined, the "Plot profile" tool of ImageJ was used to obtain the RFIs (in arbitrary units) of the parasite and associated marker, given by Channel #1 or #2, respectively, of the confocal acquisition system. This procedure can reliably estimate the level of an endolysosomal marker associated with intramacrophage *L*. *infantum*-containing PVs. Cropping of movies was performed in ImageJ (ImageJ, NIH). The percentage of intramacrophage parasites positively associated with a host cell endolysosomal marker was determined manually using Imaris software. Quantitative values obtained for host cell and parasite numbers were exported to Excel for further analysis and graphical representations. All imaging analyses and quantifications were performed blindly to eliminate any possible bias.

### Western-blot analysis

Cells are washed once with cold PBS and then treated for 15 min at 4°C with extraction buffer (25 mM HEPES, 0.5% Triton, 150 mM NaCl, 2 mM EDTA) containing proteases and phosphatase inhibitors. Protein fractions were dissolved in the appropriate volume of Laemlli buffer and incubated at 100°C for 5 min. The proteins were immediately separated on 12% SDS-polyacrylamide gels. For western-blot analysis, gels were transferred to polyvinylidene difluoride membranes (Perkin Elmer, Les Ullis, France). A primary rabbit anti-LC3 antibody was used to reveal LC3-I and LC3-II proteins. A primary mouse antibody specific for β-actin was used to verify the equal loading of lanes. Primary antibodies were revealed with anti-rabbit- or anti-mouse-peroxidase secondary antibodies. The abundance of LC3-II and β-actin proteins in the western blots was quantified by densitometry using ImageJ software.

### Quantitative real-time PCR (RT-qPCR)

Total RNA was extracted from cells using TRIzol reagent (ThermoFisher Scientific) following the manufacturer’s protocol. The level of *L*. *infantum* parasites in macrophage-like RAW 264.7 cells was quantified by *L*. *infantum* cytochrome b gene (Licytb, LinJ.02.0020) RT-qPCR in a 20-μl volume with 2 μl template DNA and 18 μl Hot FIREPol EvaGreen qPCR Mix (Solis BioDyne, Dutscher, Burmat, France) containing 1 U Taq Polymerase and 200 nM of each primer (forward [5’-CACAACAGCAAGGAGAGTGG-3’] and reverse [5’-CGATGACGAATAGTGCGAGA-3’]), designed by Primer3 web version 4.0.0 (http://primer3.ut.ee/). The amplification profile was: 94°C for 7 min, followed by 35 cycles at 94°C for 35 s, 60°C for 35 s, and 72°C for 35 s. At the end of each run, a melting curve analysis was performed from 55°C to 95°C to monitor primer dimers and verify amplicon specificity. The reactions were performed in duplicate. The level of *L*. *infantum* LEM 5700 amastigotes hosted in macrophage-like RAW 264.7 cells was quantified by amplification of the amastin gene (LinJ.34.4350), which codes for a surface glycoprotein highly expressed in the intracellular amastigote forms of *Leishmania*. Amastin RT-qPCR was performed in a 20-μl volume with 2 μl template DNA under the same conditions as those already described using specific primers (forward [5’**-**GCCGTTCTTGAGGTTGGTT**-**3]’ and reverse [5’-CGCTCGACGTGTTGATCT-T-3’]). A clinical isolate typed *L*. *infantum* MON-1, kindly provided by S. Houze (AP-HP, Bichat-Claude Bernard hospital, Paris, France), was used as an amastigote positive control and cultured promastigotes as amastigote negative controls. The *ddCt* method was used to calculate the level of total parasites and amastigote load in macrophages. Genomic DNA isolated from *L. infantum* strain MON-1 served as the quantification standard for the Licytb qPCR assay. We considered 150 ng of leishmanial DNA to be equivalent to 1.5 x 10^6^ parasites based on the conversion between the quantification of leishmanial DNA and parasites. This equivalence was used to prepare the standard curves based on serial dilutions of the *Leishmania* DNA standard in nuclease-free water, corresponding to a range of 5 × 10^7^ to 5 × 10^−3^ parasites, for which the equation was y = -1.431n(x) + 22.889 and R^2^ = 0.9958.

The determination of dynein RNA levels in non-transfected and siRNA-transfected macrophage-like RAW 264.7 cells was performed by RT-qPCR using specific dynein primers (forward [5’- TACGCTGGCTACTTTGACCA -3’] and reverse [5’-CGTTCGTCAGCATTGGAGAG-3’]). Data were normalized against that of the housekeeping gene GAPDH (forward [5’-CAAGAAGGT-GGTGAAGCAGG-3’] and reverse [5’-GCATCGAAGGTGGAAGAGTG-3’]). One microgram of extracted RNA was treated with DNase I (Amplification Grade, Life Technologies) to eliminate the risk of contamination by mouse DNA before the RT reaction was carried out using the Superscript VILO commercial kit (Life Technologies). The RT-qPCR reaction consisted of a mixture of 20 μl, including 2 μl cDNA, 10 μl SensiFAST SYBR No Rox Mix (Bioline, Paris, France) and 400 nM primers. The PCR amplification conditions consisted of an incubation cycle of 2 min at 95°C, followed by 40 cycles of amplification of 10 s at 95°C, 10 s at 60°C, and 20 s at 72°C. A melting curve cycle was performed at the end of the cycle to verify the specificity of the amplicon.

RT-qPCR determinations were monitored using the Via 7 Real-Time PCR System (ViA 7 system, ThermoFisher Scientific). The efficiency of PCR amplification was evaluated for each pair of primer and was > 1.9.

### Analysis of microtubule polymerization *in vitro*

Compounds were tested using the Tubulin Polymerization BK004P kit as specified by the manufacturer (Cytoskeleton, Inc., Denver, CO). Briefly, tubulin protein (> 99% purity) was suspended (300 μg/sample) in 100 μl G-PEM buffer (80 mM PIPES, 2 mM MgCl_2_, 0.5 mM EGTA, 1.0 mM GTP, pH 6.9) plus 5% glycerol, with or without the test compound, at 4°C. Then, the sample mixture was transferred to a pre-warmed 96-well plate and polymerization measured by the change in absorbance at 340 nm every 1 min for 60 min (SpectraMAX Plus; Molecular Devices, Inc., Sunnyvale, CA) at 37°C.

### Presentation of the data and statistics

Each experiment was performed at least two times in duplicate or triplicate. Representative confocal micrographs or movies of typical cells were used for the Fig. For the final Fig, the presented confocal micrographs were resized using Adobe Photoshop CS6 software (San Jose, CA). Quantitative data are presented as the means ± standard error of the mean (SEM). Graphs were produced using Microsoft Excel software. Comparisons between the experimental groups were performed using the unpaired Student *t*-test. Significance was established when *P* < 0.01.

## Supporting information

S1 Fig Measurement of the vacuolar space of *L*. *infantum* LEM 5700-containing tight-fitting PVs. **(A)** 3D-reconstruction surface rendering confocal micrographs showing (1) merged immunolabeling of parasite (Green channel) and CatD immunolabeling (Red channel) and (2) separated parasite immunolabeling (Green channel), and (3) separated CatD immunolabeling (Red channel). (**B**) Bar graph showing the average volumes of immunolabeled parasites and CatD immunolabeling and the volume of the vacuolar space between the parasite and PV membrane. Given that the protease localizes to the vacuolar space of PVs, the measured volume of CatD immunolabeling represents the volume of *L*. *infantum*-containing tight-fitting vacuoles. The volume of the vacuolar space between the parasite and PV membrane was calculated by subtracting the parasite volume from the vacuole volume. Confocal micrographs are representative of two independent experiments. Values were determined by analyzing at least 10 representative fusiform-like parasites. Data are presented as the average ± SEM.
(TIF)

S2 Fig Retro-2 treatment does not modify the internalization of *L*. *infantum* LEM 5700 parasites into macrophage-like RAW 264.7 cells. Bar graphs showing the number of *L*. *infantum*-infected cells (left) and *L*. *infantum* parasites per cell (right) at 0.5 h PI after the infection of cells in the continuous presence, or not, of Retro-2 (1 μM). The data are from two independent experiments. Values were determined by examining at least 50 macrophages for each condition. Data are presented as the mean ± SEM.
(TIF)

S3 Fig Distribution of vesicles positive for Stx5 in untreated and macrophage-like RAW 264.7 cells treated in the continuous presence of Retro-2. Representative 3D reconstruction CLSM micrographs showing the polarized perinuclear localization of Stx5-positive vesicles in untreated cells and their dispersion toward the cytoplasm of Retro-2 (1 μM)-treated cells. CLSM micrographs are representative of two independent experiments.
(TIF)

S4 Fig Expression and distribution of cell vesicles positive for Lamp-2 or cathepsin D in untreated and Retro-2-treated macrophage-like RAW 264.7 cells. Representative 3D reconstruction CLSM micrograph showing the unchanged cytoplasmic localization of Lamp-2- and cathepsin D (CatD)-positive vesicles in Retro-2(1 μM) -treated cells compared to untreated cells. To the right of the micrograph, bar graphs showing quantification of the Lamp-2 or CatD relative fluorescence intensity (RFI) in untreated and Retro-2-treated cells. CLSM micrographs are representative of two independent experiments. Values represent the average (± SEM) obtained by examining at least 30 infected cells per condition.
(TIF)

S5 Fig Retro-2 treatment results in failure of the heterotypic fusion between autophagosomes and lysosomes. HeLa cells stably expressing the autophagy marker microtubule-associated protein 1 light chain 3 (LC3) coupled to green fluorescent protein (GFP-LC3) were loaded with LysoTracker Red, allowing the identification and quantification of lysosomes only positive for LysoTracker Red, autophagosomes only positive for GFP-LC3, and autolysosomes positive for the merged Lysotracker Red/GFP-LC3 fluorescence signals. (**A**) *z*-stack confocal micrographs showing the rare presence of autophagosomes and the absence of autolysosomes in a control cell (left image), the classical strong presence of autolysosomes in a nutrient-starved cell (middle image), and the absence of autolysosomes, despite the presence of small and large autophagosomes, in a nutrient-starved cell subjected to the continuous presence of Retro-2 (1 μM) (right image). (**B**) Quantification of autophagosomes and autolysosomes per cell. *p < 0.01 compared to Control, **p < 0.01 compared to Autophagy-induced. (**C**) A representative western blot showing LC3 protein processing in control cells and nutrient-starved cells treated in the continuous presence of Retro-2 (1 μM), in the presence, or not, of chloroquine (CQ) (Left). Graph showing the quantification of LC3-II protein abundance (Right). *p < 0.01 compared to Control. The micrographs are representative of two independent experiments in duplicate. The white boxed areas show the region of high magnification in the adjacent images. Data were obtained by examining at least 30 cells for each condition in two independent experiments in duplicate. The western blot is representative of two separate experiments. Quantification in confocal images and western-blot quantification were performed using ImageJ software. Data are presented as the average ± SEM and were analyzed using the unpaired Student t test.
(TIF)

S6 Fig Promastigote-to-amastigote body shape differentiation of axenic *L*. *infantum* LEM 5700 parasites is unchanged in the continuous presence of Retro-2. **(A)** Micrographs showing the time-course of differentiation of axenic *L*. *infantum* parasites in the continuous presence, or not, of Retro-2 (1 μM). (**B**) Graph showing the evolution of the percentage of amastigotes during a differentiation time-course of axenic *L*. *infantum* in the continuous presence, or not, of Retro-2 (1 μM). Data were obtained from two independent experiments in duplicate. Data are presented as the mean ± SEM.
(TIF)

S1 Video Rotation of a CLSM acquisition showing the close association of Lamp-2 with *L*. *infantum* LEM 5700 parasites hosted in an infected macrophage-like RAW 264.7 cell. Video of a 3D-reconstruction surface rendering micrograph showing the merged acquisitions of immunolabeled parasites (Green, fluorescence channel 1#), immunolabeled Lamp-2 (Red, fluorescence channel 2#), and DAPI stained cell nuclei (Blue, fluorescence channel 3#). Multidimensional micrograph rotated at various angles.
(AVI)

S2 Video Rotation of a CLSM acquisition showing cathepsin D almost entirely coating *L*. *infantum* LEM 5700 parasites hosted in three infected macrophage-like RAW 264.7 cells. Video of a 3D-reconstruction surface rendering micrograph showing the merged acquisitions of immunolabeled parasites (Green, fluorescence channel 1#), immunolabeled cathepsin D (Red, fluorescence channel 2#), and DAPI stained cell nuclei (Blue, fluorescence channel 3#). Multidimensional micrograph rotated at various angles.
(MP4)

S3 Video Rotation of a CLSM acquisition showing the decreased Lamp-2 decoration of *L*. *infantum* LEM 5700 parasites hosted in five Retro-2-treated macrophage-like RAW 264.7 cells. Video of 3D-reconstruction surface rendering micrograph showing the merged acquisitions of immunolabeled parasites (Green, fluorescence channel 1#), immunolabeled Lamp-2 (Red, fluorescence channel 2#), and DAPI stained cell nuclei (Blue, fluorescence channel 3#). Multidimensional micrograph rotated at various angles.
(AVI)

S4 Video Rotation of a CLSM acquisition showing the large decrease in cathepsin D decoration of *L*. *infantum* LEM 5700 parasites hosted in a Retro-2-treated infected macrophage-like RAW 264.7 cell. Video of 3D-reconstruction surface rendering micrograph showing the merged acquisitions of immunolabeled parasites (Green, fluorescence channel 1#), immunolabeled cathepsin D (Red, fluorescence channel 2#), and DAPI stained cell nuclei (Blue, fluorescence channel 3#). Multidimensional micrograph rotated at various angles.
(MP4)

## References

[1] WHO. (2020) Global leishmaniasis surveillance, 2017–2018, and first report on 5 additional indicators Ruiz-Postigo JA, Grout L, Jaina S 95:265–280. https://www.who.int/wer/2020/wer9525/en/

[2] Alvar J, Velez ID, Bern C, Herrero M, Desjeux P, et al (2012) Leishmaniasis worldwide and global estimates of its incidence. PLoS One 7: e35671 10.1371/journal.pone.0035671 22693548 PMC3365071

[3] Podinovskaia M, Descoteaux A (2015) *Leishmania* and the macrophage: a multifaceted interaction. Future Microbiol 10: 111–129. 10.2217/fmb.14.103 25598341

[4] Young J, Kima PE (2019) The *Leishmania* parasitophorous vacuole membrane at the parasite-host interface. Yale J Biol Med 92: 511–521. 31543712 PMC6747952

[5] Horta MF, Andrade LO, Martins-Duarte ES, Castro-Gomes T (2020) Cell invasion by intracellular parasites—the many roads to infection. J Cell Sci 133. 10.1242/jcs.232488 32079731

[6] Levin R, Grinstein S, Canton J (2016) The life cycle of phagosomes: formation, maturation, and resolution. Immunol Rev 273: 156–179. 10.1111/imr.12439 27558334

[7] Desjardins M, Descoteaux A (1997) Inhibition of phagolysosomal biogenesis by the *Leishmania* lipophosphoglycan. J Exp Med 185: 2061–2068. 10.1084/jem.185.12.2061 9182677 PMC2196352

[8] Dermine JF, Goyette G, Houde M, Turco SJ, Desjardins M (2005) *Leishmania donovani* lipophosphoglycan disrupts phagosome microdomains in J774 macrophages. Cell Microbiol 7: 1263–1270. 10.1111/j.1462-5822.2005.00550.x 16098214

[9] Spath GF, Schlesinger P, Schreiber R, Beverley SM (2009) A novel role for Stat1 in phagosome acidification and natural host resistance to intracellular infection by *Leishmania major*. PLoS Pathog 5: e1000381 10.1371/journal.ppat.1000381 19381261 PMC2663844

[10] Vinet AF, Fukuda M, Turco SJ, Descoteaux A (2009) The *Leishmania donovani* lipophosphoglycan excludes the vesicular proton-ATPase from phagosomes by impairing the recruitment of synaptotagmin V. PLoS Pathog 5: e1000628 10.1371/journal.ppat.1000628 19834555 PMC2757729

[11] Lodge R, Diallo TO, Descoteaux A (2006) *Leishmania donovani* lipophosphoglycan blocks NADPH oxidase assembly at the phagosome membrane. Cell Microbiol 8: 1922–1931. 10.1111/j.1462-5822.2006.00758.x 16848789

[12] Matheoud D, Moradin N, Bellemare-Pelletier A, Shio MT, Hong WJ, et al (2013) *Leishmania* evades host immunity by inhibiting antigen cross-presentation through direct cleavage of the SNARE VAMP8. Cell Host Microbe 14: 15–25. 10.1016/j.chom.2013.06.003 23870310

[13] Alten B, Maia C, Afonso MO, Campino L, Jimenez M, et al (2016) Seasonal dynamics of phlebotomine sand fly species proven vectors of Mediterranean leishmaniasis caused by *Leishmania infantum*. PLoS Negl Trop Dis 10: e0004458 10.1371/journal.pntd.0004458 26900688 PMC4762948

[14] Ready PD (2014) Epidemiology of visceral leishmaniasis. Clin Epidemiol 6: 147–154. 10.2147/CLEP.S44267 24833919 PMC4014360

[15] Rodriguez NE, Gaur Dixit U, Allen LA, Wilson ME (2011) Stage-specific pathways of *Leishmania infantum* chagasi entry and phagosome maturation in macrophages. PLoS One 6: e19000 10.1371/journal.pone.0019000 21552562 PMC3084250

[16] Rodriguez NE, Gaur U, Wilson ME (2006) Role of caveolae in *Leishmania chagasi* phagocytosis and intracellular survival in macrophages. Cell Microbiol 8: 1106–1120. 10.1111/j.1462-5822.2006.00695.x 16819964

[17] Ueno N, Bratt CL, Rodriguez NE, Wilson ME (2009) Differences in human macrophage receptor usage, lysosomal fusion kinetics and survival between logarithmic and metacyclic *Leishmania infantum chagasi* promastigotes. Cell Microbiol 11: 1827–1841. 10.1111/j.1462-5822.2009.01374.x 19702651 PMC2788030

[18] Hsiao CH, Ueno N, Shao JQ, Schroeder KR, Moore KC, et al (2011) The effects of macrophage source on the mechanism of phagocytosis and intracellular survival of *Leishmania*. Microbes Infect 13: 1033–1044. 10.1016/j.micinf.2011.05.014 21723411 PMC3185139

[19] Eskelinen EL (2006) Roles of LAMP-1 and LAMP-2 in lysosome biogenesis and autophagy. Mol Aspects Med 27: 495–502. 10.1016/j.mam.2006.08.005 16973206

[20] Poteryaev D, Datta S, Ackema K, Zerial M, Spang A (2010) Identification of the switch in early-to-late endosome transition. Cell 141: 497–508. 10.1016/j.cell.2010.03.011 20434987

[21] Bright NA, Davis LJ, Luzio JP (2016) Endolysosomes are the principal intracellular sites of acid hydrolase activity. Curr Biol 26: 2233–2245. 10.1016/j.cub.2016.06.046 27498570 PMC5026700

[22] Kreykenbohm V, Wenzel D, Antonin W, Atlachkine V, von Mollard GF (2002) The SNAREs vti1a and vti1b have distinct localization and SNARE complex partners. Eur J Cell Biol 81: 273–280. 10.1078/0171-9335-00247 12067063

[23] Schmidt S, Joost HG, Schurmann A (2009) GLUT8, the enigmatic intracellular hexose transporter. Am J Physiol Endocrinol Metab 296: E614–618. 10.1152/ajpendo.91019.2008 19176349

[24] Schroder BA, Wrocklage C, Hasilik A, Saftig P (2010) The proteome of lysosomes. Proteomics 10: 4053–4076. 10.1002/pmic.201000196 20957757

[25] Bangert I, Tumulka F, Abele R (2011) The lysosomal polypeptide transporter TAPL: more than a housekeeping factor? Biol Chem 392: 61–66. 10.1515/BC.2011.007 21194361

[26] Dermine JF, Duclos S, Garin J, St-Louis F, Rea S, et al (2001) Flotillin-1-enriched lipid raft domains accumulate on maturing phagosomes. J Biol Chem 276: 18507–18512. 10.1074/jbc.M101113200 11279173

[27] Courret N, Frehel C, Gouhier N, Pouchelet M, Prina E, et al (2002) Biogenesis of *Leishmania*-harbouring parasitophorous vacuoles following phagocytosis of the metacyclic promastigote or amastigote stages of the parasites. J Cell Sci 115: 2303–2316. 12006615 10.1242/jcs.115.11.2303

[28] Prina E, Antoine JC, Wiederanders B, Kirschke H (1990) Localization and activity of various lysosomal proteases in *Leishmania amazonensis*-infected macrophages. Infect Immun 58: 1730–1737. 2187806 10.1128/iai.58.6.1730-1737.1990 PMC258715

[29] Stechmann B, Bai SK, Gobbo E, Lopez R, Merer G, et al (2010) Inhibition of retrograde transport protects mice from lethal ricin challenge. Cell 141: 231–242. 10.1016/j.cell.2010.01.043 20403321

[30] Forrester A, Rathjen SJ, Daniela Garcia-Castillo M, Bachert C, Couhert A, et al (2020) Functional dissection of the retrograde Shiga toxin trafficking inhibitor Retro-2. Nat Chem Biol 16: 327–336. 10.1038/s41589-020-0474-4 32080624 PMC7039708

[31] Real F, Mortara RA (2012) The diverse and dynamic nature of *Leishmania* parasitophorous vacuoles studied by multidimensional imaging. PLoS Negl Trop Dis 6: e1518 10.1371/journal.pntd.0001518 22348167 PMC3279510

[32] Scianimanico S, Desrosiers M, Dermine JF, Meresse S, Descoteaux A, et al (1999) Impaired recruitment of the small GTPase rab7 correlates with the inhibition of phagosome maturation by *Leishmania donovani* promastigotes. Cell Microbiol 1: 19–32. 10.1046/j.1462-5822.1999.00002.x 11207538

[33] Canton J, Kima PE (2012) Targeting host syntaxin-5 preferentially blocks *Leishmania* parasitophorous vacuole development in infected cells and limits experimental *Leishmania* infections. Am J Pathol 181: 1348–1355. 10.1016/j.ajpath.2012.06.041 22885104

[34] Gatica D, Lahiri V, Klionsky DJ (2018) Cargo recognition and degradation by selective autophagy. Nature Cell Biology 20: 233–242. 10.1038/s41556-018-0037-z 29476151 PMC6028034

[35] Nakamura S, Yoshimori T (2017) New insights into autophagosome-lysosome fusion. J Cell Sci 130: 1209–1216. 10.1242/jcs.196352 28302910

[36] Klionsky DJ, Abdelmohsen K, Abe A, Abedin MJ, Abeliovich H, et al (2016) Guidelines for the use and interpretation of assays for monitoring autophagy (3rd edition). Autophagy 12: 1–222. 10.1080/15548627.2015.1100356 26799652 PMC4835977

[37] Steinmetz MO, Prota AE (2018) Microtubule-targeting agents: Strategies to hijack the cytoskeleton. Trends Cell Biol 28: 776–792. 10.1016/j.tcb.2018.05.001 29871823

[38] Liu JJ (2017) Regulation of dynein-dynactin-driven vesicular transport. Traffic 18: 336–347. 10.1111/tra.12475 28248450

[39] Rai A, Pathak D, Thakur S, Singh S, Dubey AK, et al (2016) Dynein clusters into lipid microdomains on phagosomes to drive rapid transport toward lysosomes. Cell 164: 722–734. 10.1016/j.cell.2015.12.054 26853472 PMC4752818

[40] Luzio JP, Pryor PR, Bright NA (2007) Lysosomes: fusion and function. Nat Rev Mol Cell Biol 8: 622–632. 10.1038/nrm2217 17637737

[41] Desjardins M (1995) Biogenesis of phagolysosomes: the 'kiss and run' hypothesis. Trends Cell Biol 5: 183–186. 10.1016/s0962-8924(00)88989-8 14731444

[42] Marques ARA, Saftig P (2019) Lysosomal storage disorders—challenges, concepts and avenues for therapy: beyond rare diseases. J Cell Sci 132. 10.1242/jcs.221739 30651381

[43] Blocker A, Griffiths G, Olivo JC, Hyman AA, Severin FF (1998) A role for microtubule dynamics in phagosome movement. J Cell Sci 111 (Pt 3): 303–312. 9427679 10.1242/jcs.111.3.303

[44] Harrison RE, Bucci C, Vieira OV, Schroer TA, Grinstein S (2003) Phagosomes fuse with late endosomes and/or lysosomes by extension of membrane protrusions along microtubules: role of Rab7 and RILP. Mol Cell Biol 23: 6494–6506. 10.1128/mcb.23.18.6494-6506.2003 12944476 PMC193691

[45] Keller S, Berghoff K, Kress H (2017) Phagosomal transport depends strongly on phagosome size. Sci Rep 7: 17068 10.1038/s41598-017-17183-7 29213131 PMC5719076

[46] Lang T, Hellio R, Kaye PM, Antoine JC (1994) *Leishmania donovani*-infected macrophages: characterization of the parasitophorous vacuole and potential role of this organelle in antigen presentation. J Cell Sci 107: 2137–2150. 7983173 10.1242/jcs.107.8.2137

[47] Dermine JF, Scianimanico S, Prive C, Descoteaux A, Desjardins M (2000) *Leishmania* promastigotes require lipophosphoglycan to actively modulate the fusion properties of phagosomes at an early step of phagocytosis. Cell Microbiol 2: 115–126. 10.1046/j.1462-5822.2000.00037.x 11207568

[48] Mittra B, Andrews NW (2013) IRONy OF FATE: role of iron-mediated ROS in *Leishmania* differentiation. Trends Parasitol 29: 489–496. 10.1016/j.pt.2013.07.007 23948431 PMC3783550

[49] Spath GF, Drini S, Rachidi N (2015) A touch of Zen: post-translational regulation of the *Leishmania* stress response. Cell Microbiol 17: 632–638. 10.1111/cmi.12440 25801803

[50] Kima PE (2007) The amastigote forms of *Leishmania* are experts at exploiting host cell processes to establish infection and persist. Int J Parasitol 37: 1087–1096. 10.1016/j.ijpara.2007.04.007 17543969 PMC2043126

[51] McConville MJ, de Souza D, Saunders E, Likic VA, Naderer T (2007) Living in a phagolysosome; metabolism of *Leishmania* amastigotes. Trends Parasitol 23: 368–375. 10.1016/j.pt.2007.06.009 17606406

[52] Canton J, Kima PE (2012) Interactions of pathogen-containing compartments with the secretory pathway. Cell Microbiol 14: 1676–1686. 10.1111/cmi.12000 22862745

[53] McConville MJ, Naderer T (2011) Metabolic pathways required for the intracellular survival of *Leishmania*. Annu Rev Microbiol 65: 543–561. 10.1146/annurev-micro-090110-102913 21721937

[54] McConville MJ (2016) Metabolic crosstalk between *Leishmania* and the macrophage host. Trends Parasitol 32: 666–668. 10.1016/j.pt.2016.05.005 27234812

[55] McConville MJ, Saunders EC, Kloehn J, Dagley MJ (2015) *Leishmania* carbon metabolism in the macrophage phagolysosome- feast or famine? F1000 Res 4: 938. 10.12688/f1000research.6724.1 PMC4648189 26594352

[56] Gupta N, Noel R, Goudet A, Hinsinger K, Michau A, et al (2017) Inhibitors of retrograde trafficking active against ricin and Shiga toxins also protect cells from several viruses, *Leishmania* and *Chlamydiales*. Chem Biol Interact 267: 96–103. 10.1016/j.cbi.2016.10.005 27712998

[57] Nonnenmacher ME, Cintrat JC, Gillet D, Weber T (2015) Syntaxin 5-dependent retrograde transport to the trans-Golgi network is required for adeno-associated virus transduction. J Virol 89: 1673–1687. 10.1128/JVI.02520-14 25410859 PMC4300741

[58] Lipovsky A, Popa A, Pimienta G, Wyler M, Bhan A, et al (2013) Genome-wide siRNA screen identifies the retromer as a cellular entry factor for human papillomavirus. Proc Natl Acad Sci U S A 110: 7452–7457. 10.1073/pnas.1302164110 23569269 PMC3645514

[59] Morgens DW, Chan C, Kane AJ, Weir NR, Li A, et al (2019) Retro-2 protects cells from ricin toxicity by inhibiting ASNA1-mediated ER targeting and insertion of tail-anchored proteins. Elife 8. 10.7554/eLife.48434 PMC6858068 31674906

[60] Dai W, Wu Y, Bi J, Lu X, Hou A, et al (2017) Antiviral effects of Retro-2(cycl) and Retro-2.1 against Enterovirus 71 in vitro and in vivo. Antiviral Res 144: 311–321. 10.1016/j.antiviral.2017.07.001 28688753

[61] Shtanko O, Sakurai Y, Reyes AN, Noel R, Cintrat JC, et al (2018) Retro-2 and its dihydroquinazolinone derivatives inhibit filovirus infection. Antiviral Res 149: 154–163. 10.1016/j.antiviral.2017.11.016 29175127

[62] Reck-Peterson SL, Redwine WB, Vale RD, Carter AP (2018) The cytoplasmic dynein transport machinery and its many cargoes. Nat Rev Mol Cell Biol 19: 382–398. 10.1038/s41580-018-0004-3 29662141 PMC6457270

[63] Delevoye C, Goud B (2015) Rab GTPases and kinesin motors in endosomal trafficking. Methods Cell Biol 130: 235–246. 10.1016/bs.mcb.2015.05.004 26360038

[64] Farhan H (2020) Rendezvous of Retro-2 at the ER. Nat Chem Biol 16: 229–230. 10.1038/s41589-020-0475-3 32080623

[65] Hancock WO (2014) Bidirectional cargo transport: moving beyond tug of war. Nat Rev Mol Cell Biol 15: 615–628. 10.1038/nrm3853 25118718 PMC5014371

[66] Janke C, Bulinski JC (2011) Post-translational regulation of the microtubule cytoskeleton: mechanisms and functions. Nat Rev Mol Cell Biol 12: 773–786. 10.1038/nrm3227 22086369

[67] Canton J, Ndjamen B, Hatsuzawa K, Kima PE (2012) Disruption of the fusion of *Leishmania* parasitophorous vacuoles with ER vesicles results in the control of the infection. Cell Microbiol 14: 937–948. 10.1111/j.1462-5822.2012.01767.x 22309219

[68] Craig E, Huyghues-Despointes CE, Yu C, Handy EL, Sello JK, et al (2017) Structurally optimized analogs of the retrograde trafficking inhibitor Retro-2cycl limit *Leishmania* infections. PLoS Negl Trop Dis 11: e0005556 10.1371/journal.pntd.0005556 28505157 PMC5444862

[69] Descoteaux A (2018) The macrophage–parasite interface as a chemotherapeutic target in leishmaniasis In: Rivas L, Gil C, editors. Drug discovery for leishmaniasis. London: The Royal Society of Chemistry pp. 387–395.

[70] Lamotte S, Spath GF, Rachidi N, Prina E (2017) The enemy within: Targeting host-parasite interaction for antileishmanial drug discovery. PLoS Negl Trop Dis 11: e0005480 10.1371/journal.pntd.0005480 28594938 PMC5464532

[71] Jain K, Jain NK (2013) Novel therapeutic strategies for treatment of visceral leishmaniasis. Drug Discov Today 18: 1272–1281. 10.1016/j.drudis.2013.08.005 23973338

[72] Lang T, de Chastellier C, Frehel C, Hellio R, Metezeau P, et al (1994) Distribution of MHC class I and of MHC class II molecules in macrophages infected with *Leishmania amazonensis*. J Cell Sci 107: 69–82. 8175924 10.1242/jcs.107.1.69

[73] Augustin R, Riley J, Moley KH (2005) GLUT8 contains a [DE]XXXL[LI] sorting motif and localizes to a late endosomal/lysosomal compartment. Traffic 6: 1196–1212. 10.1111/j.1600-0854.2005.00354.x 16262729

[74] Wolters JC, Abele R, Tampe R (2005) Selective and ATP-dependent translocation of peptides by the homodimeric ATP binding cassette transporter TAP-like (ABCB9). J Biol Chem 280: 23631–23636. 10.1074/jbc.M503231200 15863492

[75] Bosc D, Mouray E, Cojean S, Franco CH, Loiseau PM, et al (2016) Highly improved antiparasitic activity after introduction of an N-benzylimidazole moiety on protein farnesyltransferase inhibitors. Eur J Med Chem 109: 173–186. 10.1016/j.ejmech.2015.12.045 26774924

[76] Bampton ET, Goemans CG, Niranjan D, Mizushima N, Tolkovsky AM (2005) The dynamics of autophagy visualized in live cells: from autophagosome formation to fusion with endo/lysosomes. Autophagy 1: 23–36. 10.4161/auto.1.1.1495 16874023

