# Supplementary figures and images for: The macrophage microtubule network acts as a key cellular controller of the intracellular fate of Leishmania infantum
Source: PLoS Negl Trop Dis. 2020 Jul 28;14(7):e0008396. doi: 10.1371/journal.pntd.0008396 (PMC7386624; doi:10.1371/journal.pntd.0008396)

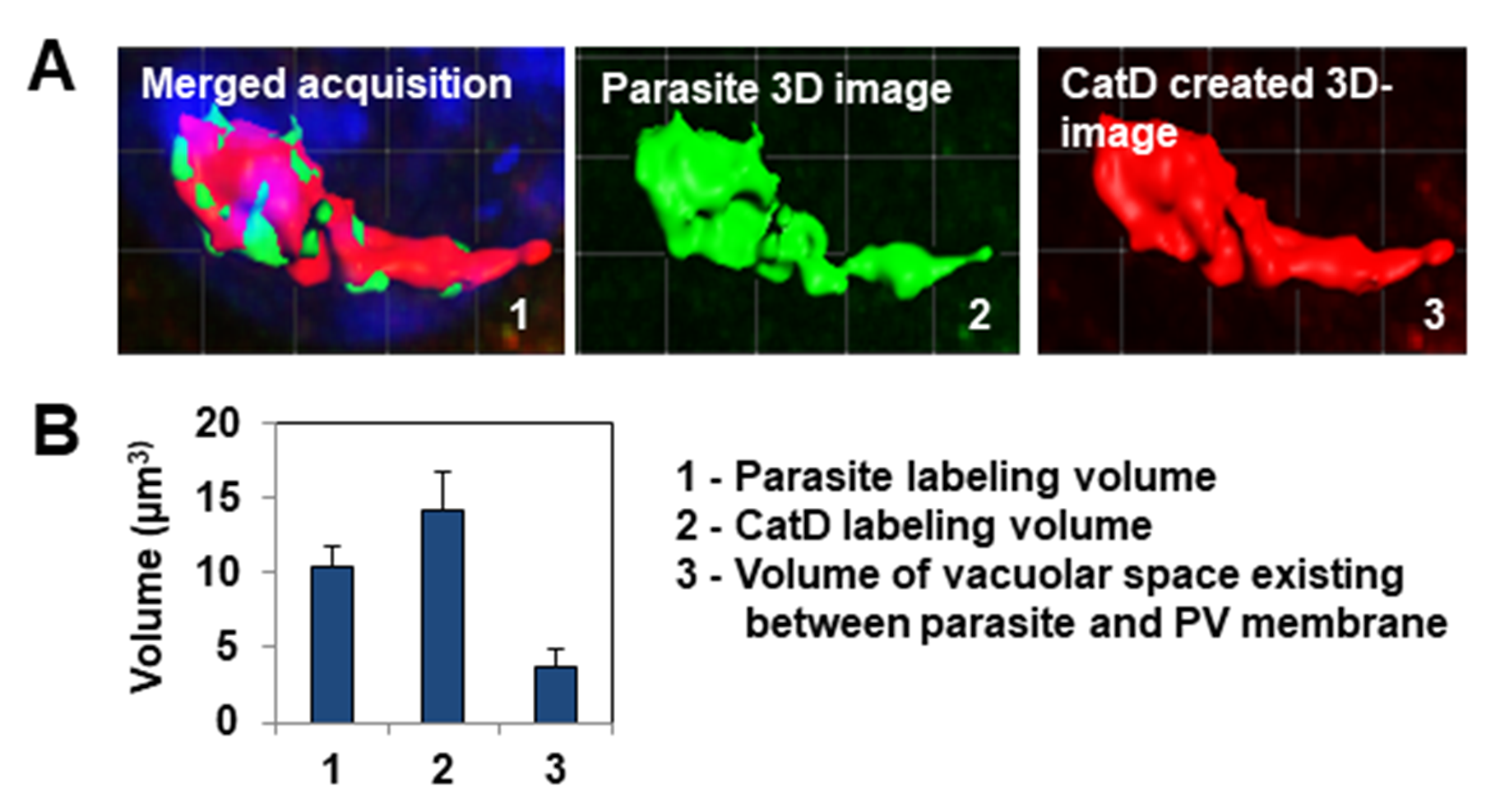

Supplement: S1 Fig — (A) 3D-reconstruction surface rendering confocal micrographs showing (1) merged immunolabeling of parasite (Green channel) and CatD immunolabeling (Red channel) and (2) separated parasite immunolabeling (Green channel), and (3) separated CatD immunolabeling (Red channel). (B) Bar graph showing the average volumes of immunolabeled parasites and CatD immunolabeling and the volume of the vacuolar space between the parasite and PV membrane. Given that the protease localizes to the vacuolar space of PVs, the measured volume of CatD immunolabeling represents the volume of L. infantum-containing tight-fitting vacuoles. The volume of the vacuolar space between the parasite and PV membrane was calculated by subtracting the parasite volume from the vacuole volume. Confocal micrographs are representative of two independent experiments. Values were determined by analyzing at least 10 representative fusiform-like parasites. Data are presented as the average ± SEM. (TIF) [file pntd.0008396.s001.tif]

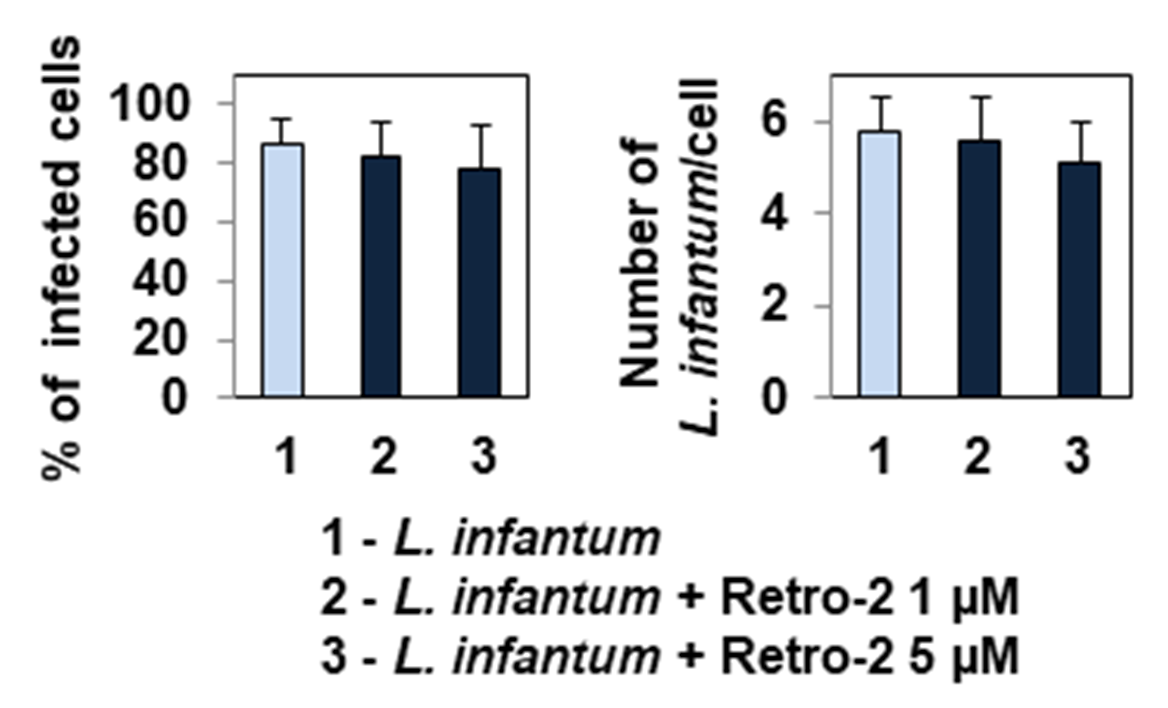

Supplement: S2 Fig — Bar graphs showing the number of L. infantum-infected cells (left) and L. infantum parasites per cell (right) at 0.5 h PI after the infection of cells in the continuous presence, or not, of Retro-2 (1 μM). The data are from two independent experiments. Values were determined by examining at least 50 macrophages for each condition. Data are presented as the mean ± SEM. (TIF) [file pntd.0008396.s002.tif]

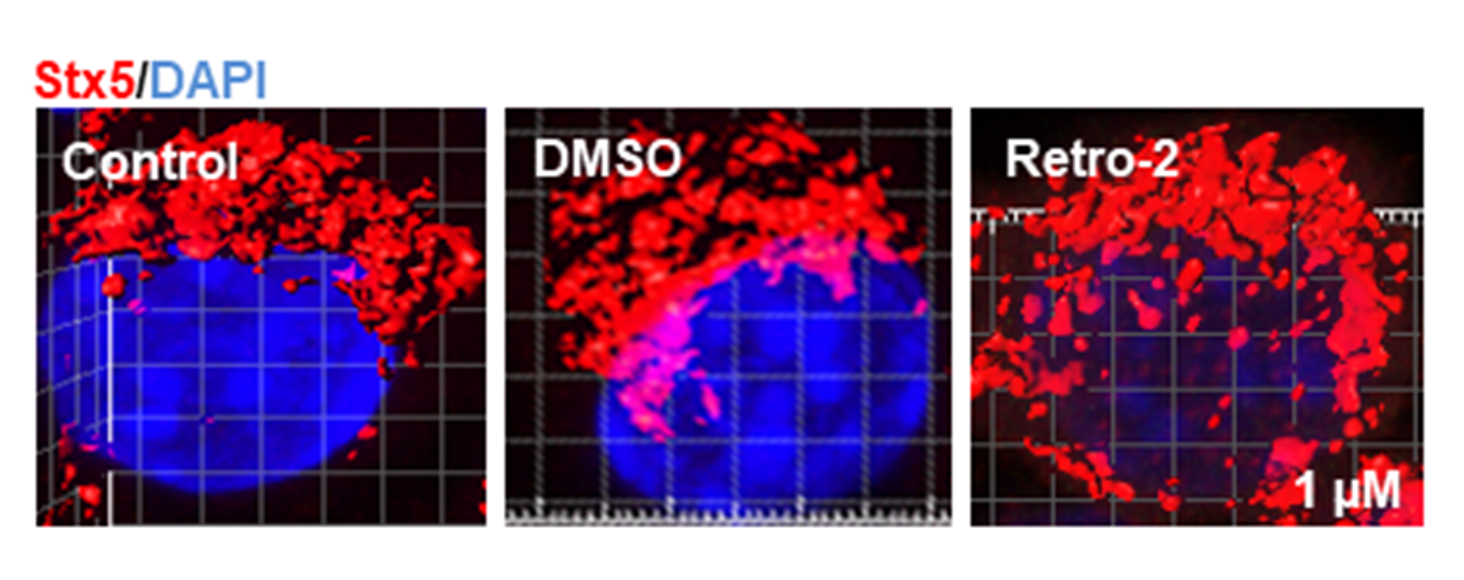

Supplement: S3 Fig — Representative 3D reconstruction CLSM micrographs showing the polarized perinuclear localization of Stx5-positive vesicles in untreated cells and their dispersion toward the cytoplasm of Retro-2 (1 μM)-treated cells. CLSM micrographs are representative of two independent experiments. (TIF) [file pntd.0008396.s003.tif]

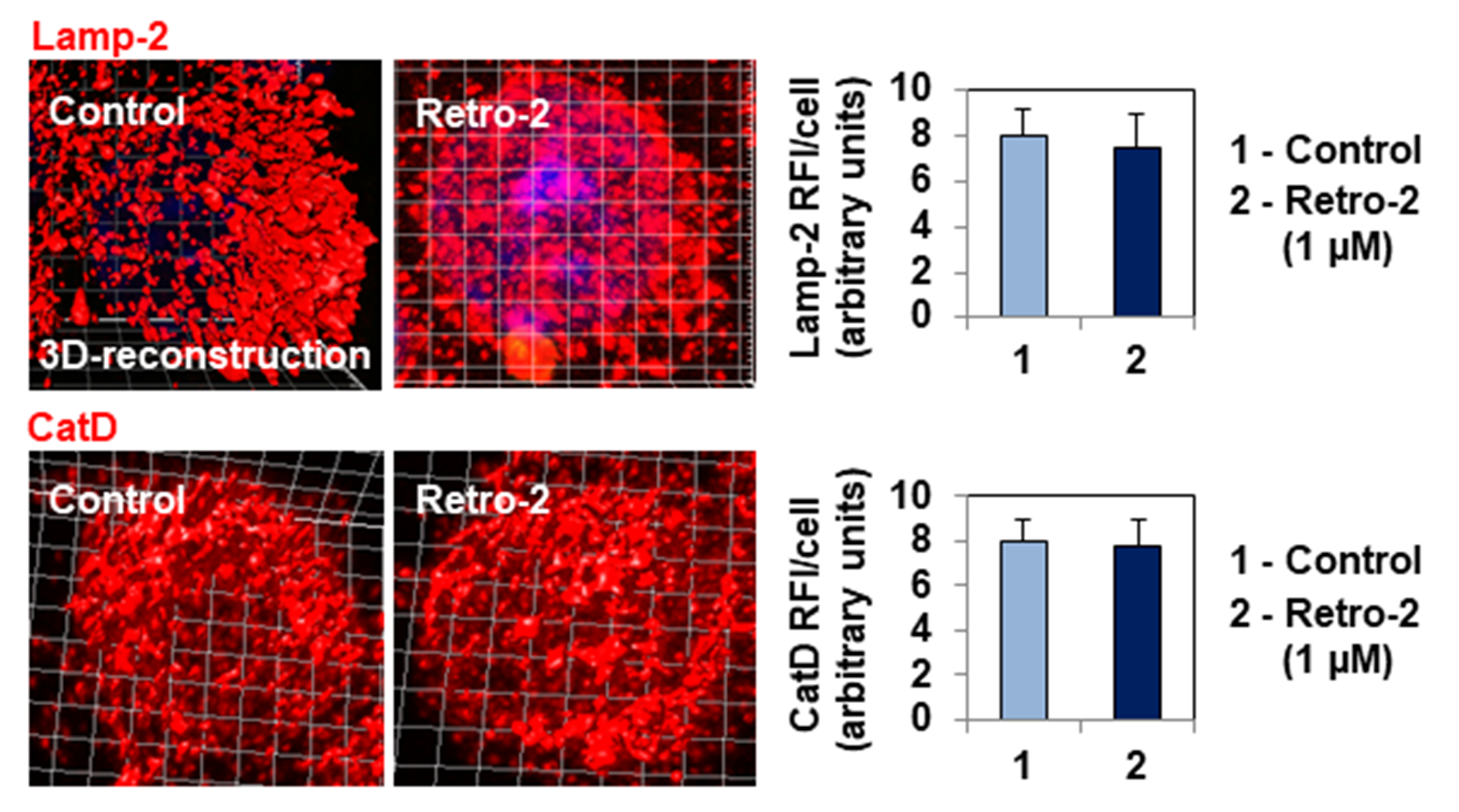

Supplement: S4 Fig — Representative 3D reconstruction CLSM micrograph showing the unchanged cytoplasmic localization of Lamp-2- and cathepsin D (CatD)-positive vesicles in Retro-2(1 μM) -treated cells compared to untreated cells. To the right of the micrograph, bar graphs showing quantification of the Lamp-2 or CatD relative fluorescence intensity (RFI) in untreated and Retro-2-treated cells. CLSM micrographs are representative of two independent experiments. Values represent the average (± SEM) obtained by examining at least 30 infected cells per condition. (TIF) [file pntd.0008396.s004.tif]

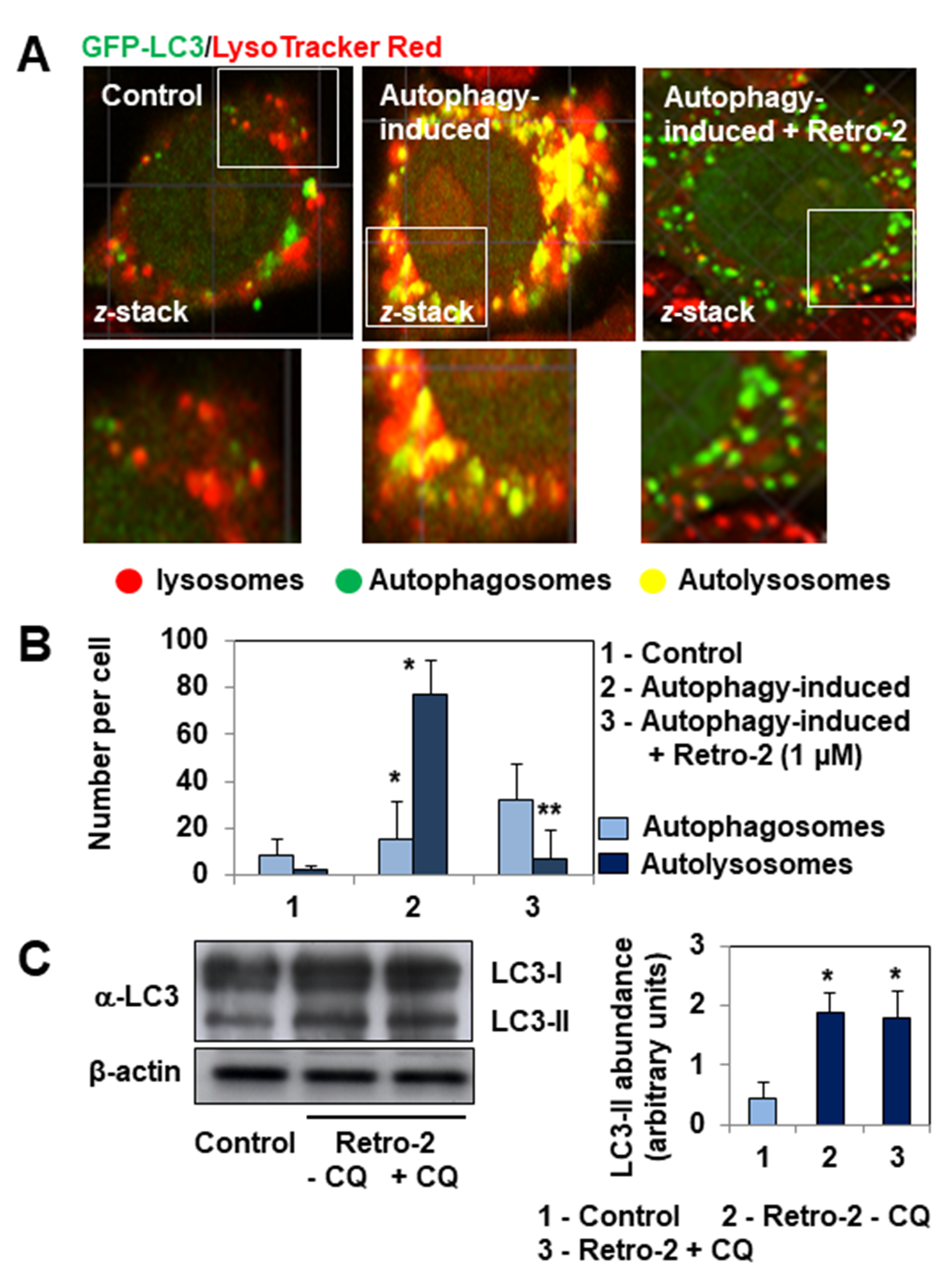

Supplement: S5 Fig — HeLa cells stably expressing the autophagy marker microtubule-associated protein 1 light chain 3 (LC3) coupled to green fluorescent protein (GFP-LC3) were loaded with LysoTracker Red, allowing the identification and quantification of lysosomes only positive for LysoTracker Red, autophagosomes only positive for GFP-LC3, and autolysosomes positive for the merged Lysotracker Red/GFP-LC3 fluorescence signals. (A) z-stack confocal micrographs showing the rare presence of autophagosomes and the absence of autolysosomes in a control cell (left image), the classical strong presence of autolysosomes in a nutrient-starved cell (middle image), and the absence of autolysosomes, despite the presence of small and large autophagosomes, in a nutrient-starved cell subjected to the continuous presence of Retro-2 (1 μM) (right image). (B) Quantification of autophagosomes and autolysosomes per cell. *p < 0.01 compared to Control, **p < 0.01 compared to Autophagy-induced. (C) A representative western blot showing LC3 protein processing in control cells and nutrient-starved cells treated in the continuous presence of Retro-2 (1 μM), in the presence, or not, of chloroquine (CQ) (Left). Graph showing the quantification of LC3-II protein abundance (Right). *p < 0.01 compared to Control. The micrographs are representative of two independent experiments in duplicate. The white boxed areas show the region of high magnification in the adjacent images. Data were obtained by examining at least 30 cells for each condition in two independent experiments in duplicate. The western blot is representative of two separate experiments. Quantification in confocal images and western-blot quantification were performed using ImageJ software. Data are presented as the average ± SEM and were analyzed using the unpaired Student t test. (TIF) [file pntd.0008396.s005.tif]

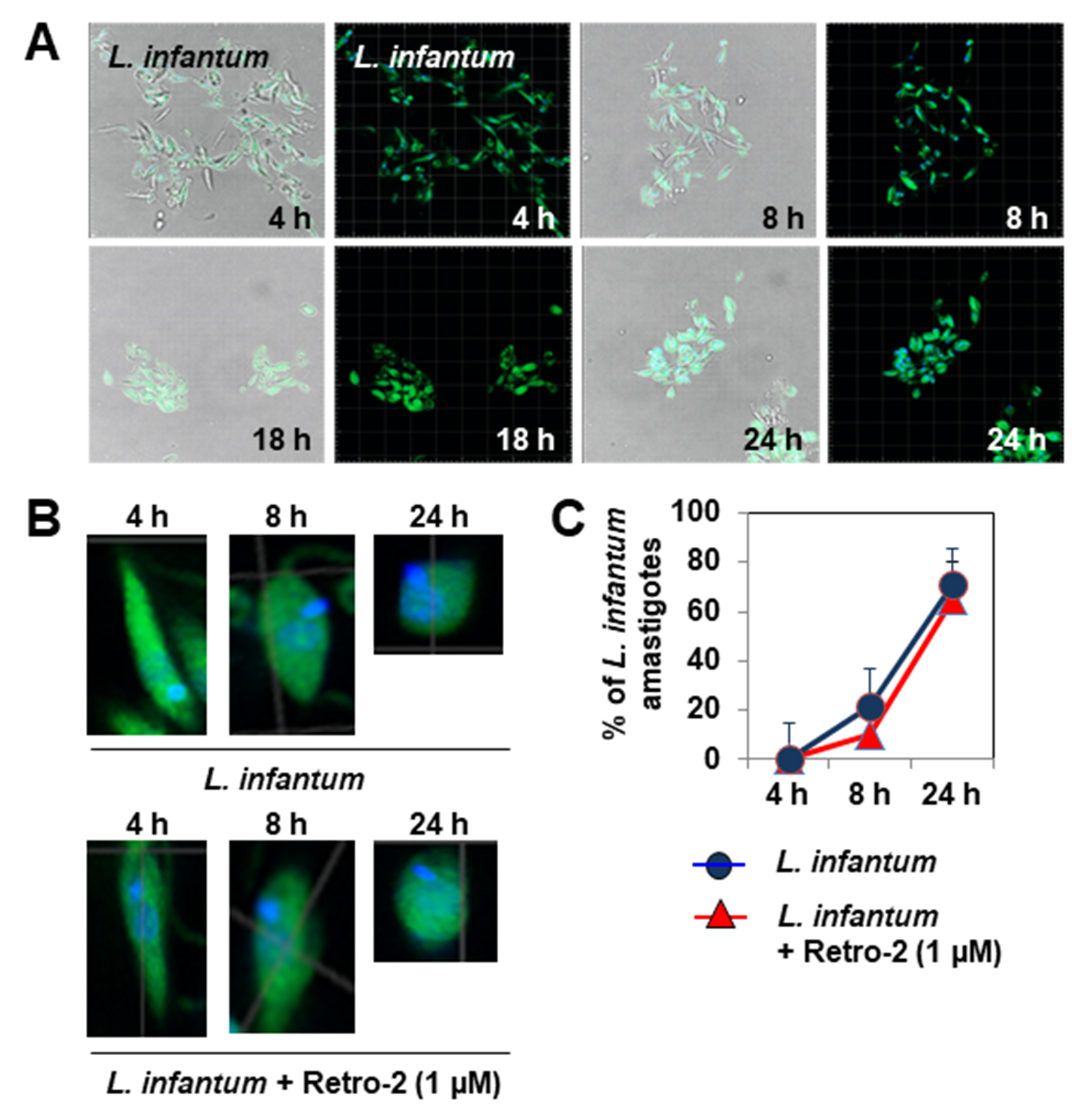

Supplement: S6 Fig — (A) Micrographs showing the time-course of differentiation of axenic L. infantum parasites in the continuous presence, or not, of Retro-2 (1 μM). (B) Graph showing the evolution of the percentage of amastigotes during a differentiation time-course of axenic L. infantum in the continuous presence, or not, of Retro-2 (1 μM). Data were obtained from two independent experiments in duplicate. Data are presented as the mean ± SEM. (TIF) [file pntd.0008396.s006.tif]
